# Supplementary figures and images for: Tracing the evolution and genomic dynamics of mating-type loci in Cryptococcus pathogens and closely related species
Source: bioRxiv. 2025 Aug 30:2025.02.12.637874. Originally published 2025 Feb 16. Preprint. [Version 2] doi: 10.1101/2025.02.12.637874 (PMC11844451; doi:10.1101/2025.02.12.637874)

**A****Cryptococcus****Kwoniella**

SH-aLRT / UFboot

gCF | sCF

0.1

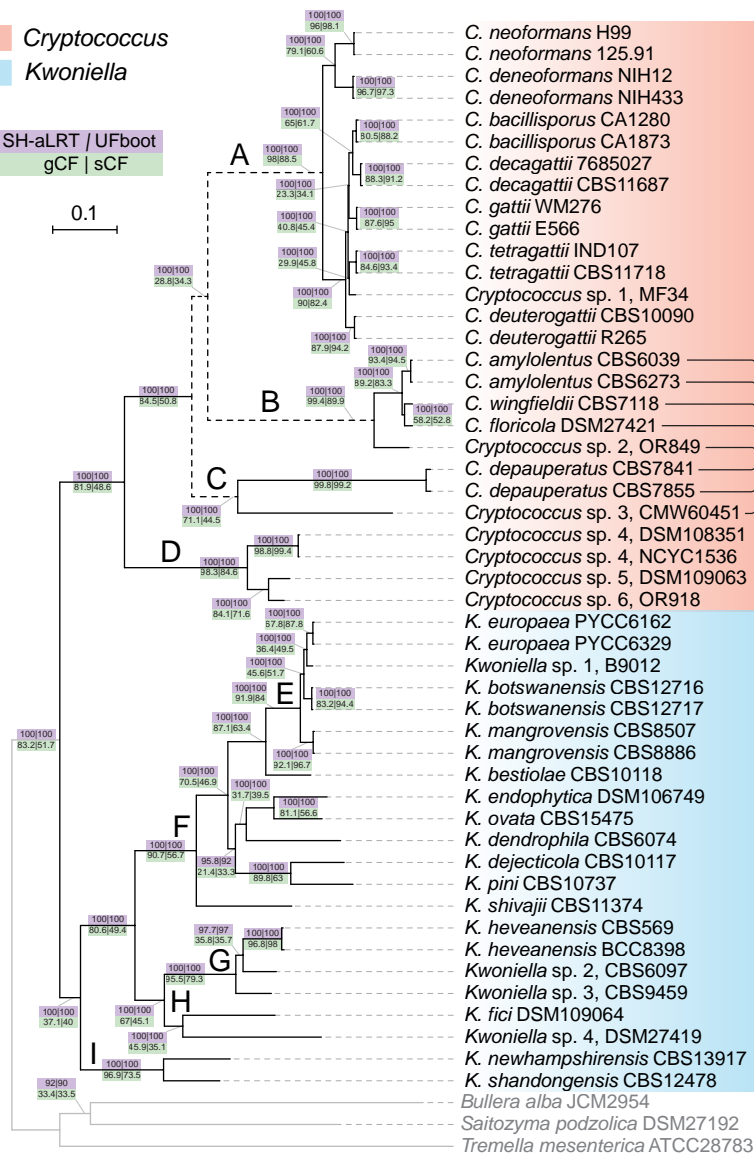**B**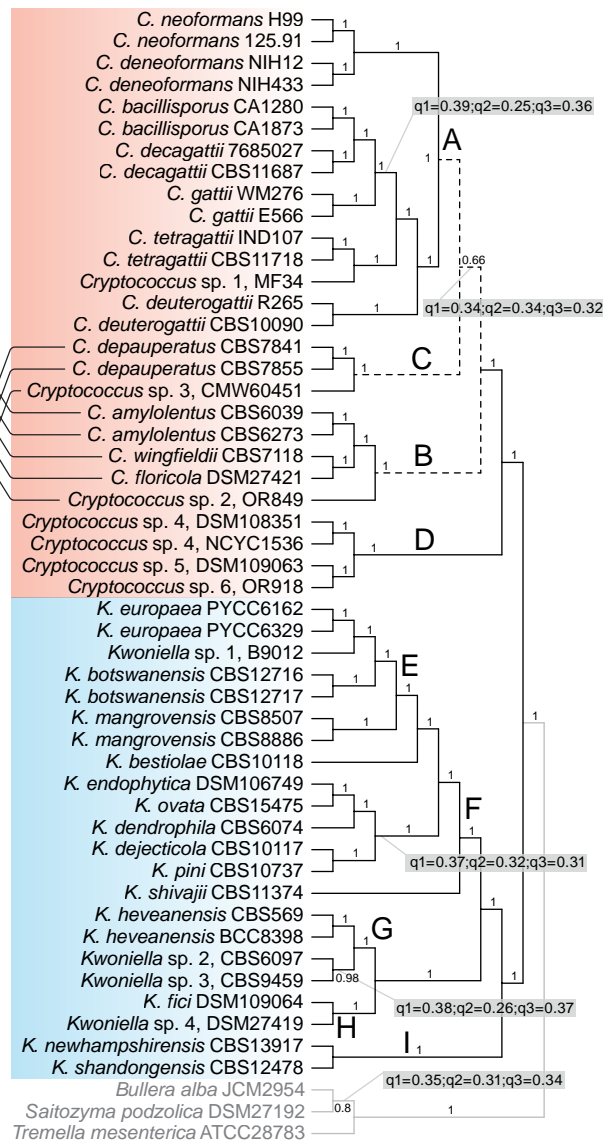

Supplement: Supplement 1 — S1 Fig. Cryptococcus and Kwoniella species phylogenies. Both trees were inferred using a dataset of 3,086 single-copy genes shared across all species and three outgroups (depicted in grey). (A) Phylogeny inferred using a concatenation-based approach, corresponding to the tree in Fig 1A, but with all branch support and concordance values displayed (SH-aLRT, UFBoot, gCF, and sCF). (B) Tree topology inferred using a coalescence-based approach (ASTRAL), with local posterior probability (LPP) values shown for each branch. Quartet values for the main topology (q1) and alternative topologies (q2 and q3) are included for branches where q1 < 0.4, reflecting some uncertainty considering the expected value of 0.33 for a hard polytomy. [file media-1.pdf]

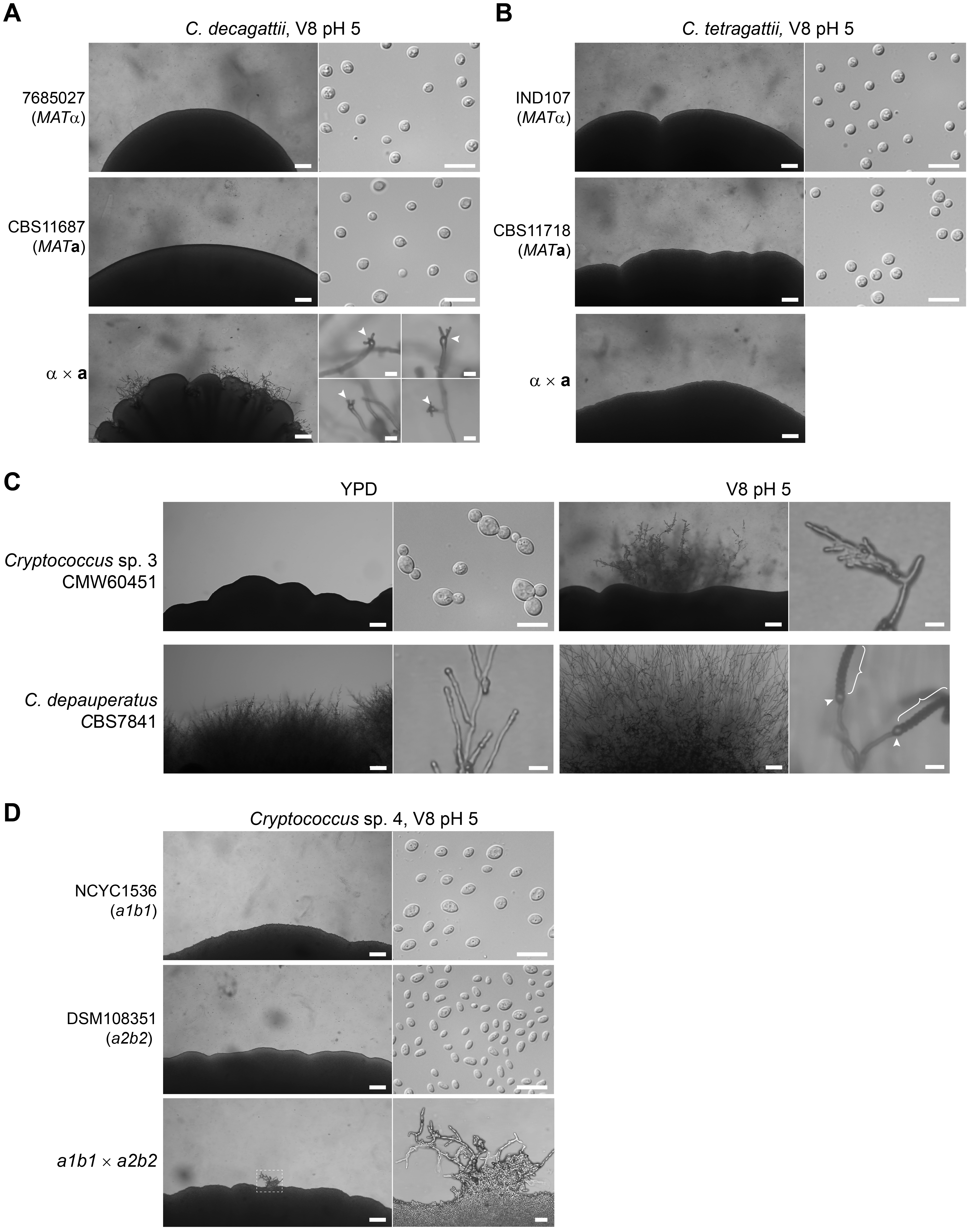

Supplement: Supplement 2 — S2 Fig. Assessing sexual reproduction assays in Cryptococcus species. All micrographs are from cultures incubated for 2 weeks in the dark at room temperature on the indicated media. For each panel, the left image shows the colony edge, and the right image shows a higher-magnification view of cell or hyphal morphology from the same culture. (A) C. decagattii: solo cultures of strains 7685027 (MATα) and CBS11687 (MATa) produced only yeast cells (right panels). In contrast, the α × a cross generated hyphae at the colony margin, with basidia and a few elongated basidiospores (arrowheads). Long spore chains were not observed, possibly due to rapid spore discharge. (B) C. tetragattii: solo cultures of strains IND107 (MATα) and CBS11718 (MATa) produced only yeast cells (right panels), and the α × a cross did not form hyphae or sexual structures, even after prolonged incubation (>1 month). (C) Solo cultures of Cryptococcus sp. 3 (CMW60451) and C. depauperatus (CBS7841) on YPD and V8 pH 5 media. C. depauperatus produced abundant hyphae on both media; however, basidia (arrowhead) and spore chains (brackets) were only observed under V8 conditions. In contrast, Cryptococcus sp. 3 grew primarily as yeast on YPD, occasionally forming pseudohyphae on V8 pH 5, but no sexual structures were detected even after extended incubation (>1 month). (D) Cryptococcus sp. 4: solo cultures of NCYC1536 (a1b1) and DSM108351 (a2b2) grew as yeast (right panels). The a1b1 × a2b2 cross produced limited hyphae (right panel, zoomed view), but these were incipient and non-recurrent, and no sexual structures were observed even after >2 months. Scale bars: 200 μm (colony edge images); 10 μm (right panels), except for panel D bottom right = 25 μm. [file media-2.jpg]

**A**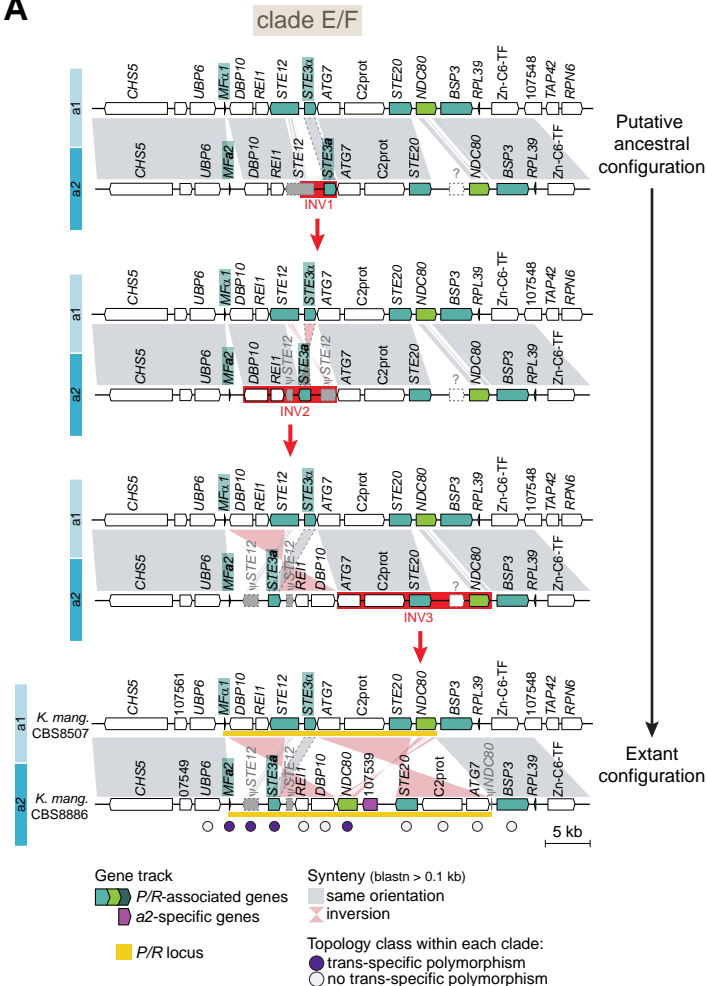**B**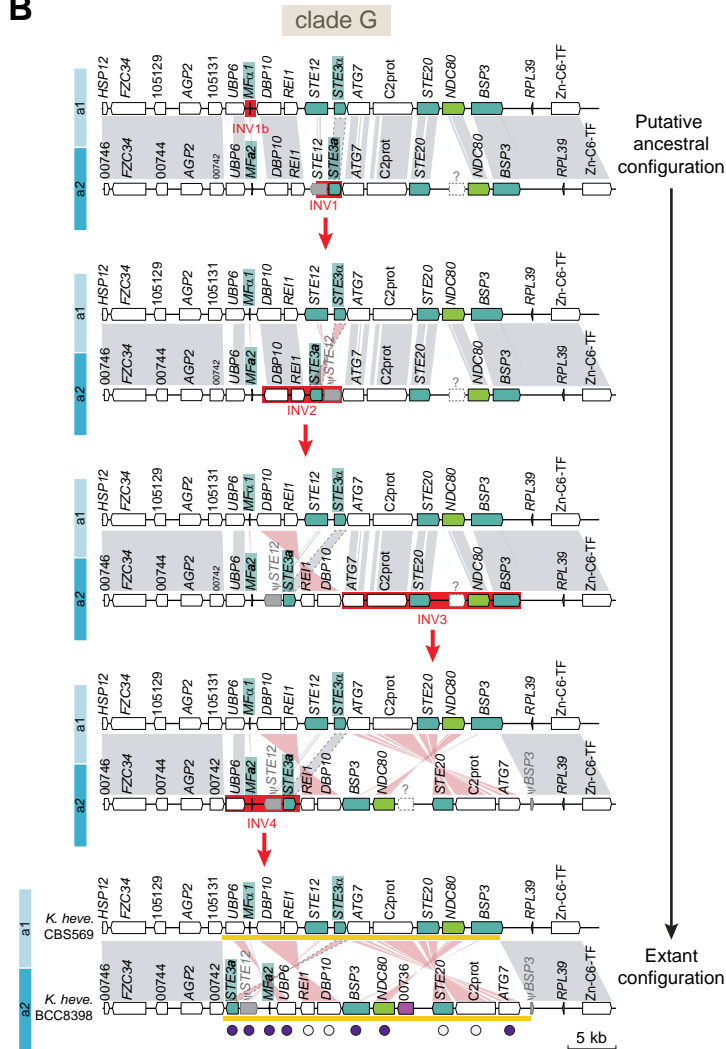**C**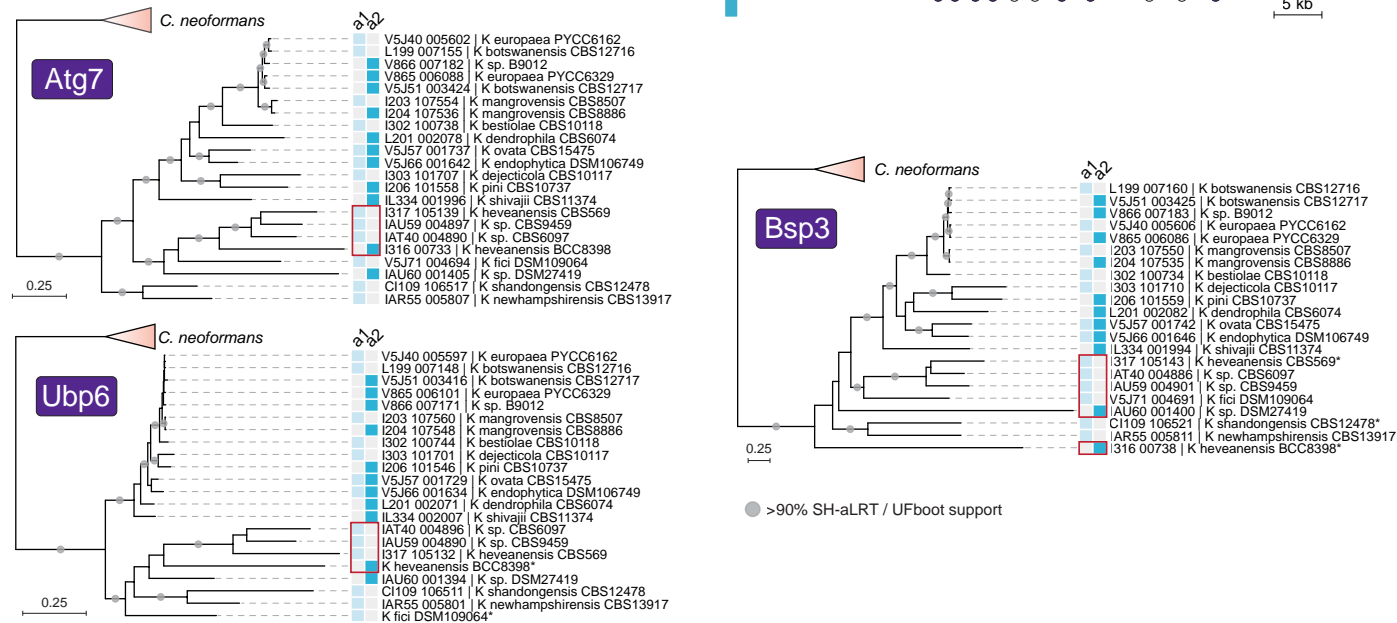

Supplement: Supplement 5 — S5 Fig. Predicted rearrangements leading to the extant P/R configuration in Kwoniella species within clades E/F and clade G. (A) The extant P/R locus configuration in K. mangrovensis is inferred to derive from a putative ancestral state through three inversion events: (INV1) involving STE3 and STE12, truncating STE12 in the a2 allele; (INV2) relocating DBP10 and REI1 from the locus edge to its center; and (INV3) moving NDC80 from the edge to the middle of the locus. (B) The extant P/R locus configuration in K. heveanensis, inferred from a putative ancestral state involving four inversion events: (INV1) involving STE3 and STE12, truncating STE12 in the a2 allele; (INV1b) inverting only the pheromone gene; (INV2) relocating DBP10 and REI1 from the locus edge to its center; (INV3) relocating BSP3-NDC80 to the middle of the locus; and (INV4) relocating UBP6 to the center of the locus. INV1 and INV2 are likely ancestral in Kwoniella. (C) Gene genealogies reveal trans-specific polymorphism for ATG7, UBP6, and BSP3 within clade G species only. Genes that required manual correction are marked with an asterisk. [file media-5.pdf]

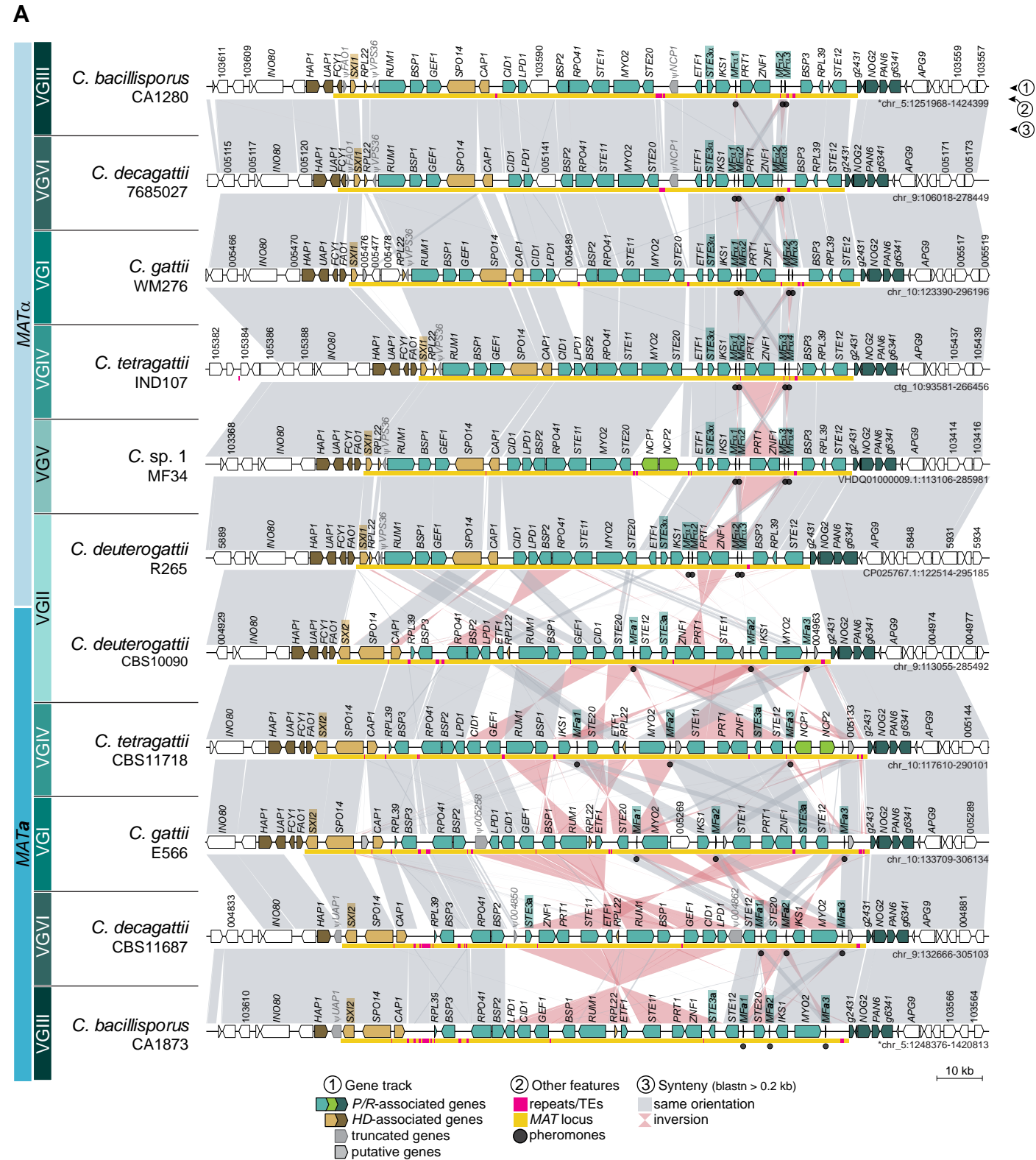

**B**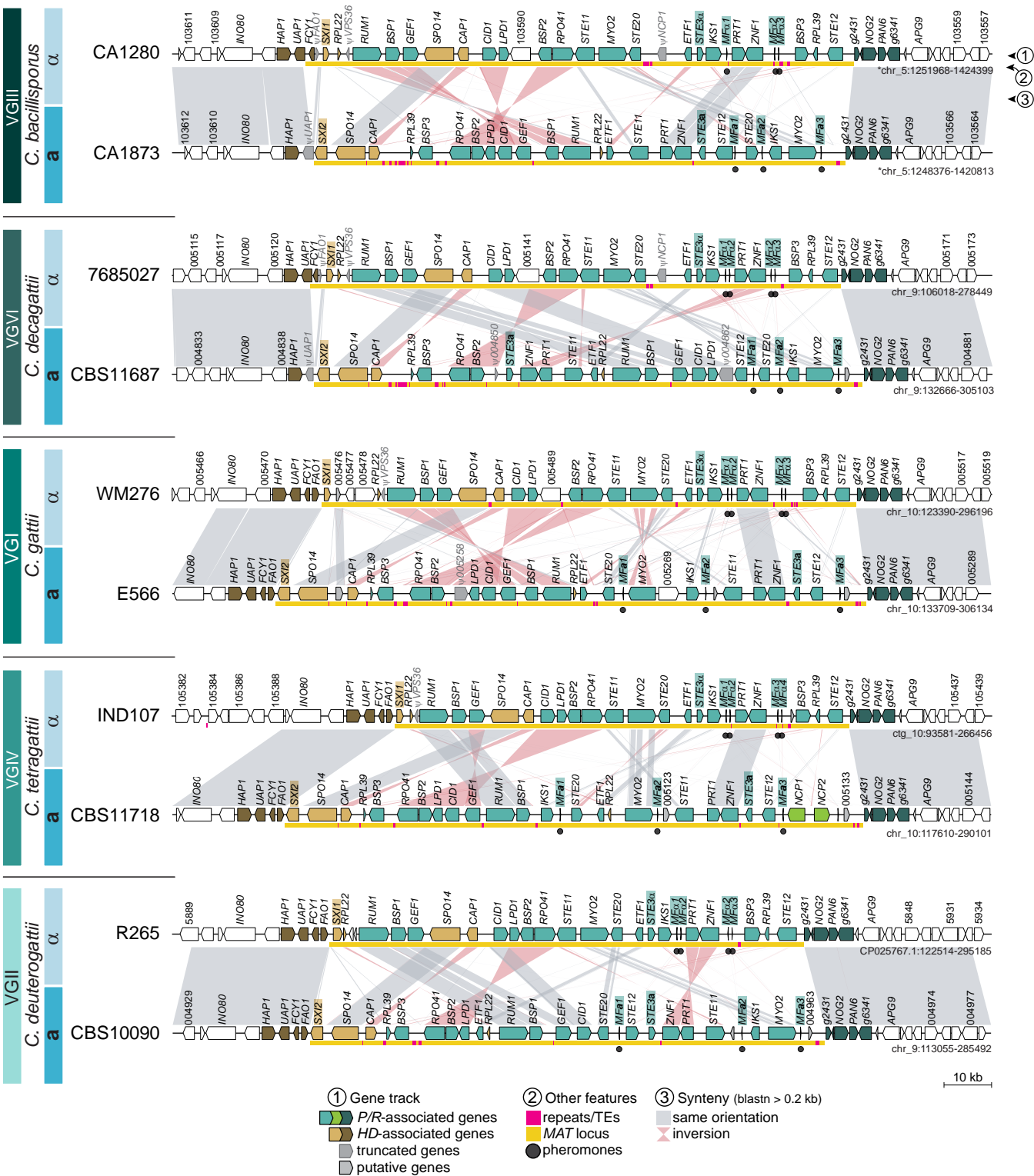

Supplement: Supplement 7 — S7 Fig. MAT locus structure in Cryptococcus gattii species complex. This supplementary figure spans 2 pages. (A) Synteny analysis with strains organized by mating type and, within each mating type, sorted according to their phylogenetic relationships. The analysis shows that the MATa configuration is highly variable across species, with each species exhibiting a distinct organization. In contrast, the MATα structure is more conserved across species. (B) Synteny analysis with strains grouped by species to compare differences between mating types and define the exact boundaries of the MAT locus based on synteny conservation. Note that no MATa strain of the VGV lineage has been isolated to date. In both panels, chromosomes inverted relative to their original assembly orientations are marked with asterisks. [file media-7.pdf]

Bsp3

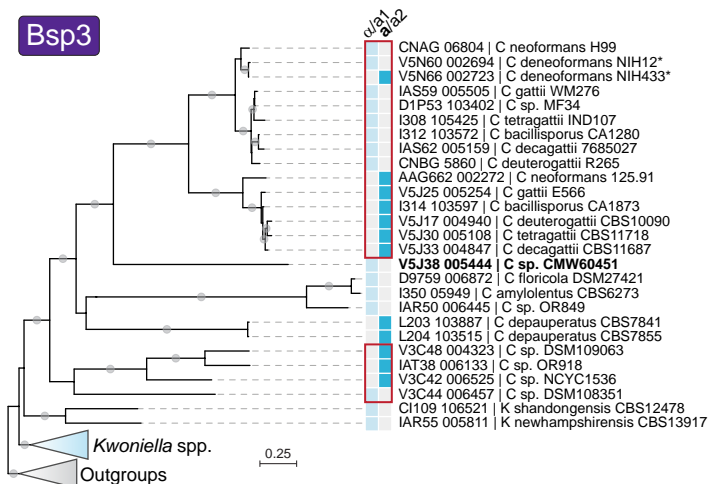

Prt1

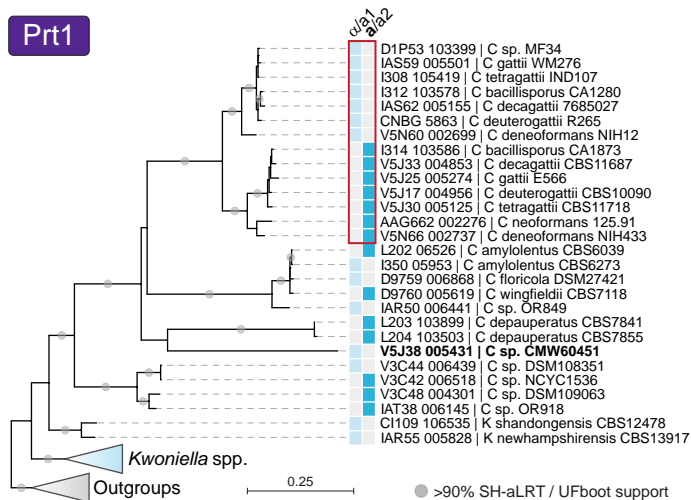

Spo14

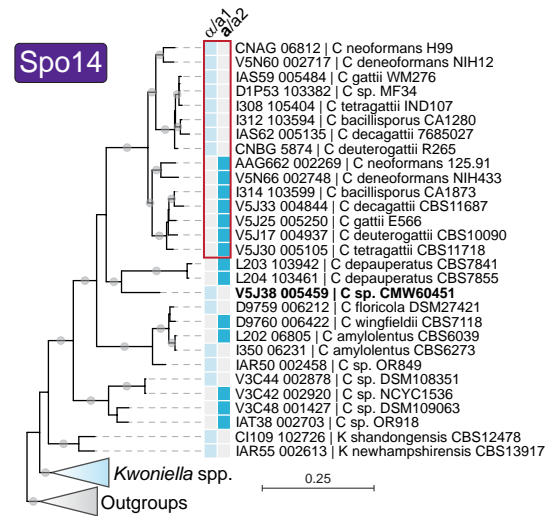

Cap1

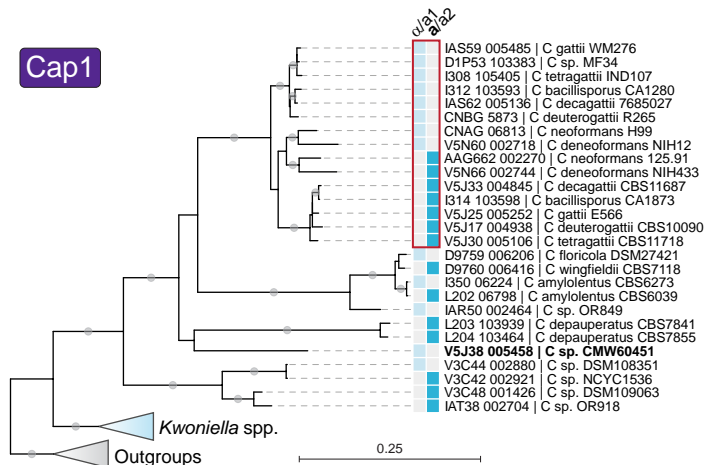

Supplement: Supplement 8 — S8 Fig. Phylogenies of BSP3, PRT1, SPO14, and CAP1 within the predicted MAT locus of Cryptococcus sp. 3. In pathogenic Cryptococcus species, these genes exhibit trans-specific polymorphism, characterized by mating-type-specific clustering, with a and α alleles forming distinct clades. An exception is observed in BSP3 from C. deneoformans (marked with asterisks), where mating-type specificity has been lost, representing a derived state. In contrast, protein sequences from Cryptococcus sp. 3 (highlighted in bold) do not group with either the a or α allele-specific clusters and instead occupy distinct positions outside these groups. Maximum likelihood phylogenies were constructed using protein sequences. For clarity, sequences from other Cryptococcus and Kwoniella species are collapsed, and well-supported nodes (≥90% SH-aLRT/UFBoot support) are indicated by filled circles. Scale bars represent the number of substitutions per site. [file media-8.pdf]

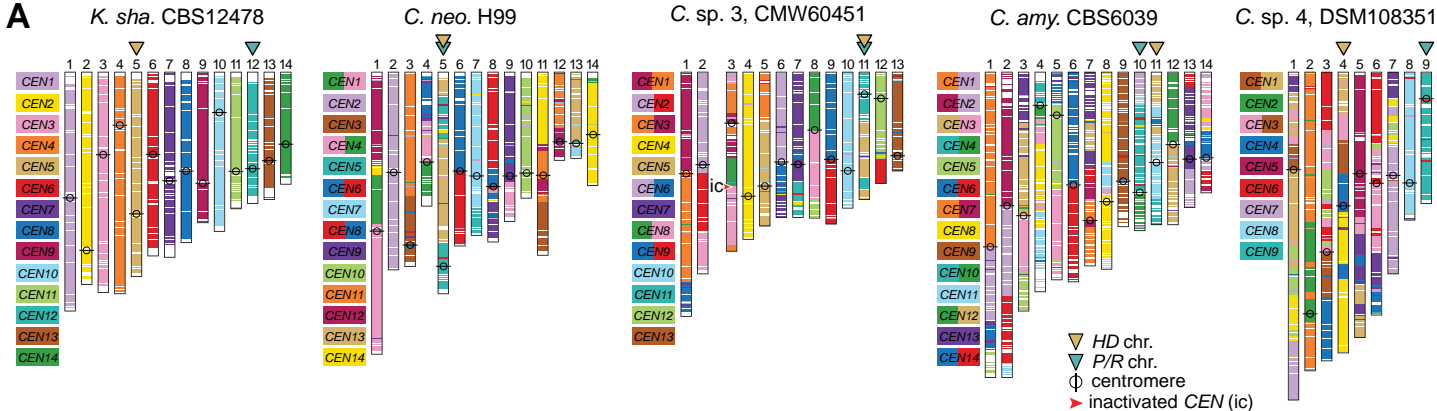

**B**

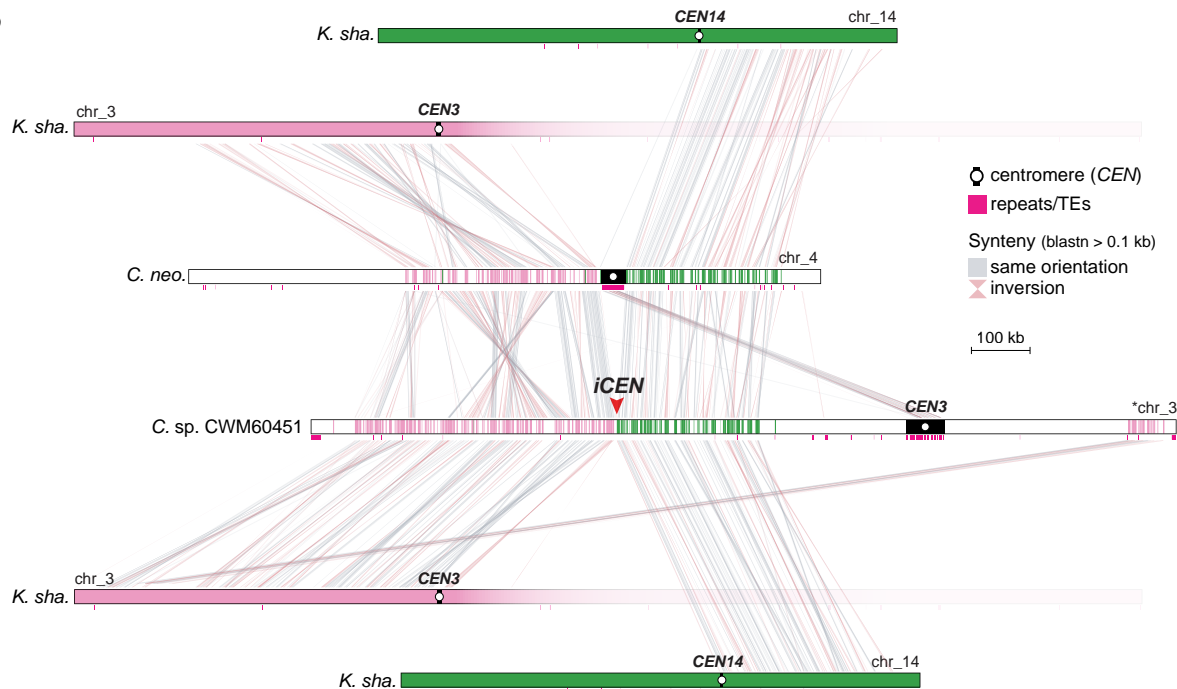

Supplement: Supplement 9 — S9 Fig. Chromosome number reduction in Cryptococcus sp. 3 (CWM60451) involves centromere inactivation via loss of LTR-rich regions. (A) The karyotype of K. shandongensis (with 14 chrs.) served as the reference for reconstructing synteny blocks in pairwise comparisons with representative Cryptococcus species of clades A (C. neoformans H99), B (C. amylolentus CBS6039), C (Cryptococcus sp. CMW60451), and D (Cryptococcus sp. DSM108351). Each K. shandongensis chromosome is assigned a distinct color, which defines the color scheme for homologous synteny blocks in the corresponding chromosomes of other species. Centromeres are labeled “CEN” and colored according to their associated reference chromosome. Centromeres inferred to result from intercentromeric recombination are shown with two colors, reflecting ancestry from two distinct K. shandongensis centromeres. Inactivated centromeres (“iCEN” or “ic”) are marked with red arrowheads. (B) Linear chromosome plots show synteny conservation among these species and detail the region of centromere inactivation (iCEN) in Cryptococcus sp. 3. Centromeres are marked with black boxes with white circles and repeat-rich regions are highlighted in pink. [file media-9.pdf]

cell cluster A

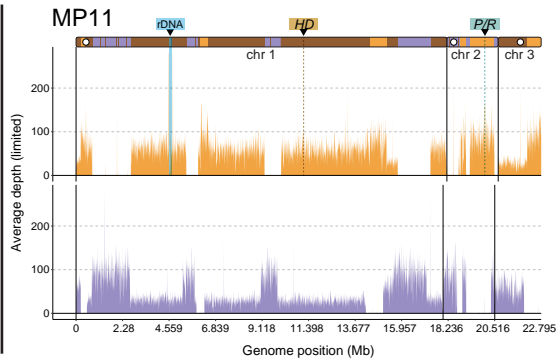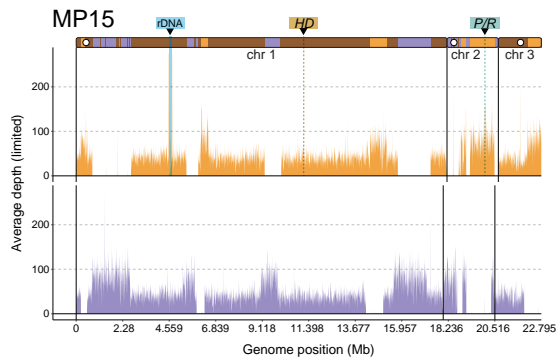

cell cluster C

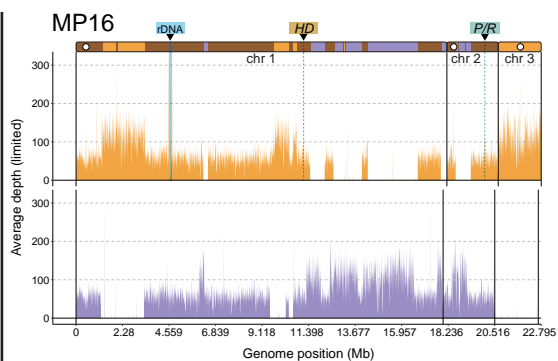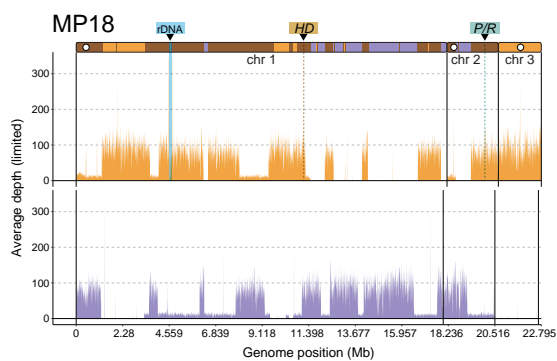

cell cluster G

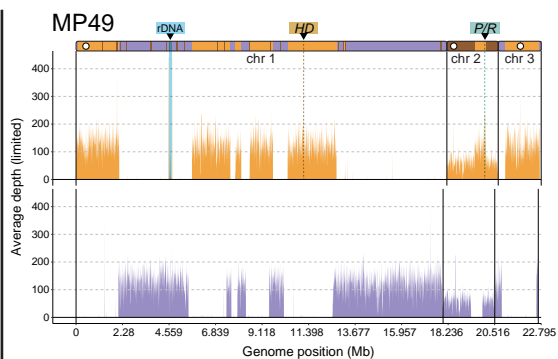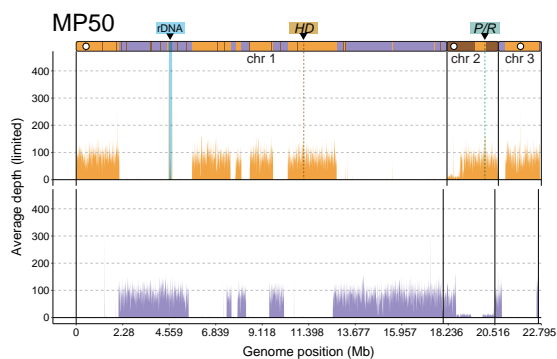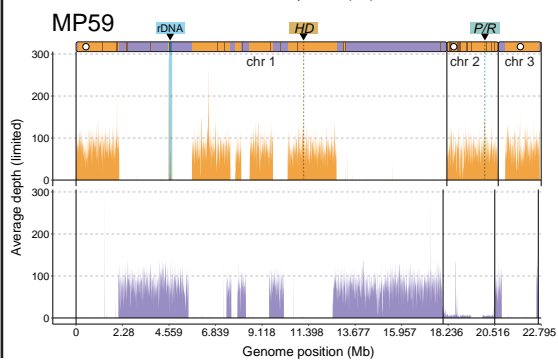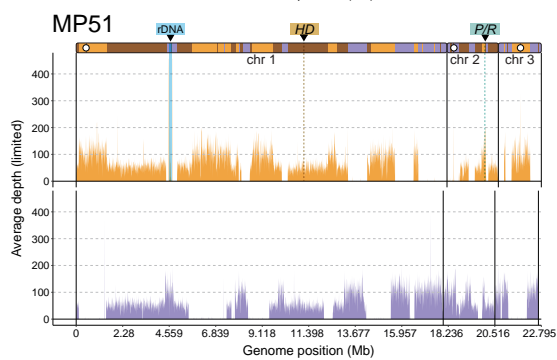

cell cluster H

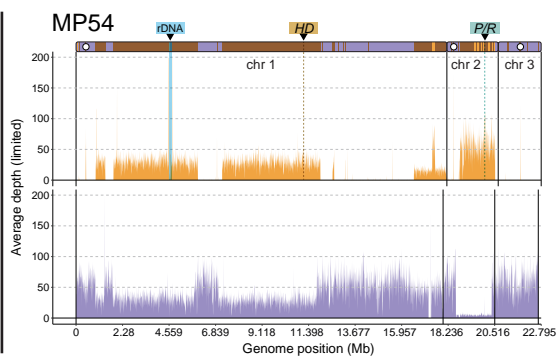

■ CBS8507 (P1)  
■ CBS10435 (P2)  
■ heterozygous  
 ◻ centromere

Supplement: Supplement 10 — S10 Fig. Nuclear genome composition of K. mangrovensis progeny reveals substantial aneuploidy and recombination. Sequencing read-depth coverage and inheritance patterns of progeny derived from a CBS8507 (a1b1) × CBS10435 (a2b2) sexual cross. For each progeny, sequencing coverage plots (normalized to the genome-wide average coverage) are color-coded according to each parent contribution, as shown in the key. Haplotypes blocks inferred from SNP data are overlaid for each chromosome for comparison, reflecting instances of recombination or loss of heterozygosity (LOH). In some strains (MP18, MP50, MP59 and MP54), coverage analysis suggests potential changes in ploidy in a subset of the sequenced cell population. This is indicated by skewed read proportions favoring one parent genome over the other while still retaining the corresponding haplotype, suggestive of genomic instability. [file media-10.pdf]

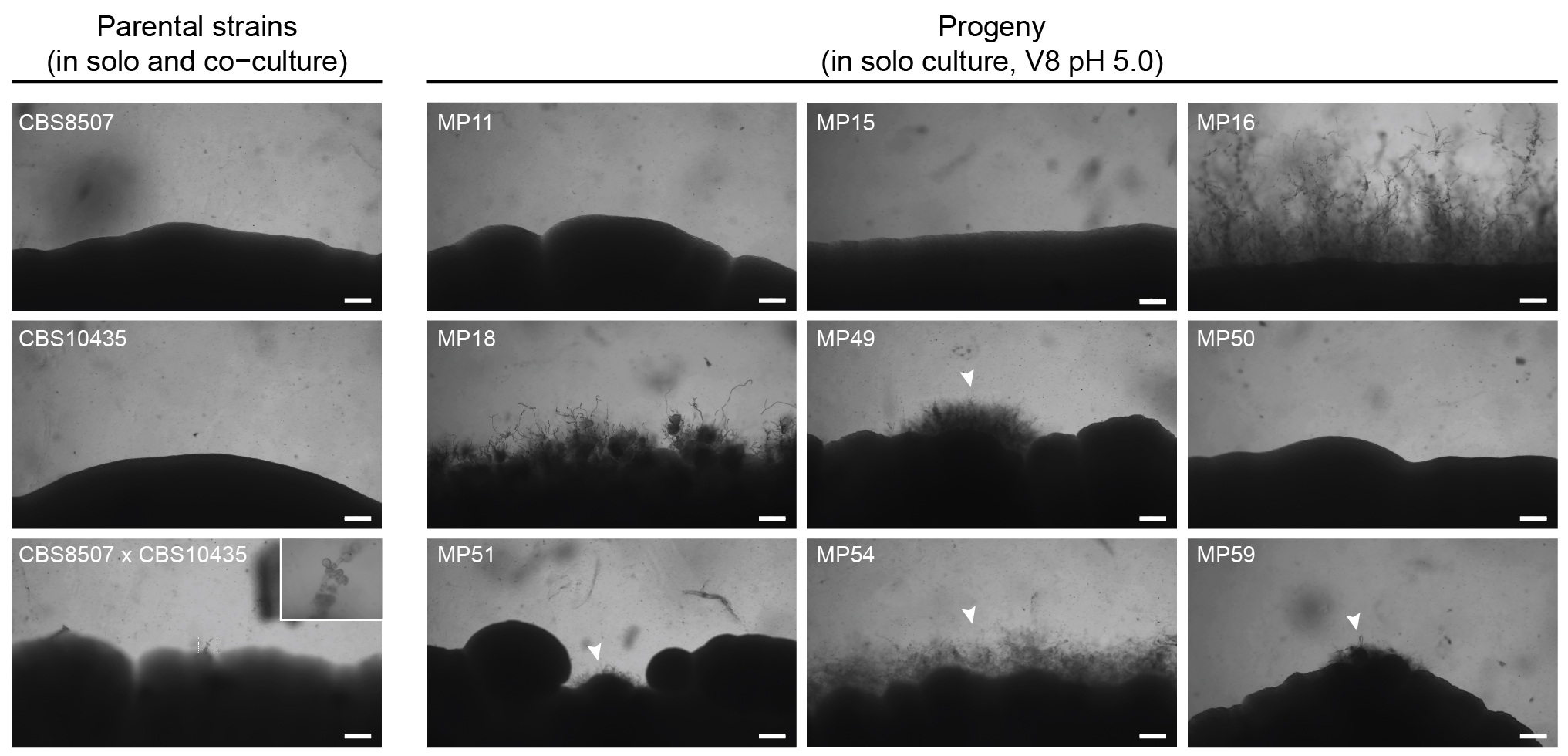

Supplement: Supplement 12 — S12 Fig. Self-filamentation phenotype of K. mangrovensis progeny. Parental strains CBS8507 and CBS10435 (grown individually and in co-culture) and their recovered progeny were cultivated on V8, pH 5.0 at room temperature. Self-filamentation was assessed after 2 weeks of incubation. While neither parental strain exhibited self-filamentation in solo culture, their co-culture produced hyphal filaments and basidia. [file media-12.jpg]
