# Supplementary material for: Tracing the evolution and genomic dynamics of mating-type loci in Cryptococcus pathogens and closely related species
Source: bioRxiv. 2025 Aug 30:2025.02.12.637874. Originally published 2025 Feb 16. Preprint. [Version 2] doi: 10.1101/2025.02.12.637874 (PMC11844451; doi:10.1101/2025.02.12.637874)
Supplement: Supplement 3 — S3 Fig. Structure and genomic context of the HD mating-type locus in tetrapolar Cryptococcus and Kwoniella species. This supplementary figure spans five pages and contains a total of 15 panels (labeled A–O), with three consecutive panels per page. For each group of three panels, the top-left panel shows a synteny view of the full chromosomes, highlighting the chromosomal location of the HD locus; the bottom panel provides a zoomed-in synteny view (~200 kb) centered on the HD locus; and the top-right panel displays a simplified phylogenetic tree, providing contextual information for the species included in the synteny analyses. For cross-referencing, HD-associated genes in the zoomed-in panel are colored gold if the corresponding ortholog in Cryptococcus pathogens is located within the MAT locus or shown in a darker shade when positioned in the immediate flanking regions. The P/R allele of each strain (a1 or a2) is indicated on the left. Chromosomes inverted relative to their original assembly orientations are marked with asterisks. In panel G, only one representative of K. europaea and K. botswanensis is shown, and in panel M, only one representative of K. heveanensis is included, as the genomes of their mating-type counterparts are not assembled at the chromosome level; the scaffolds containing the HD locus are, however, included in the zoomed-in view. Other features are annotated as shown in the key. [file media-3.pdf]

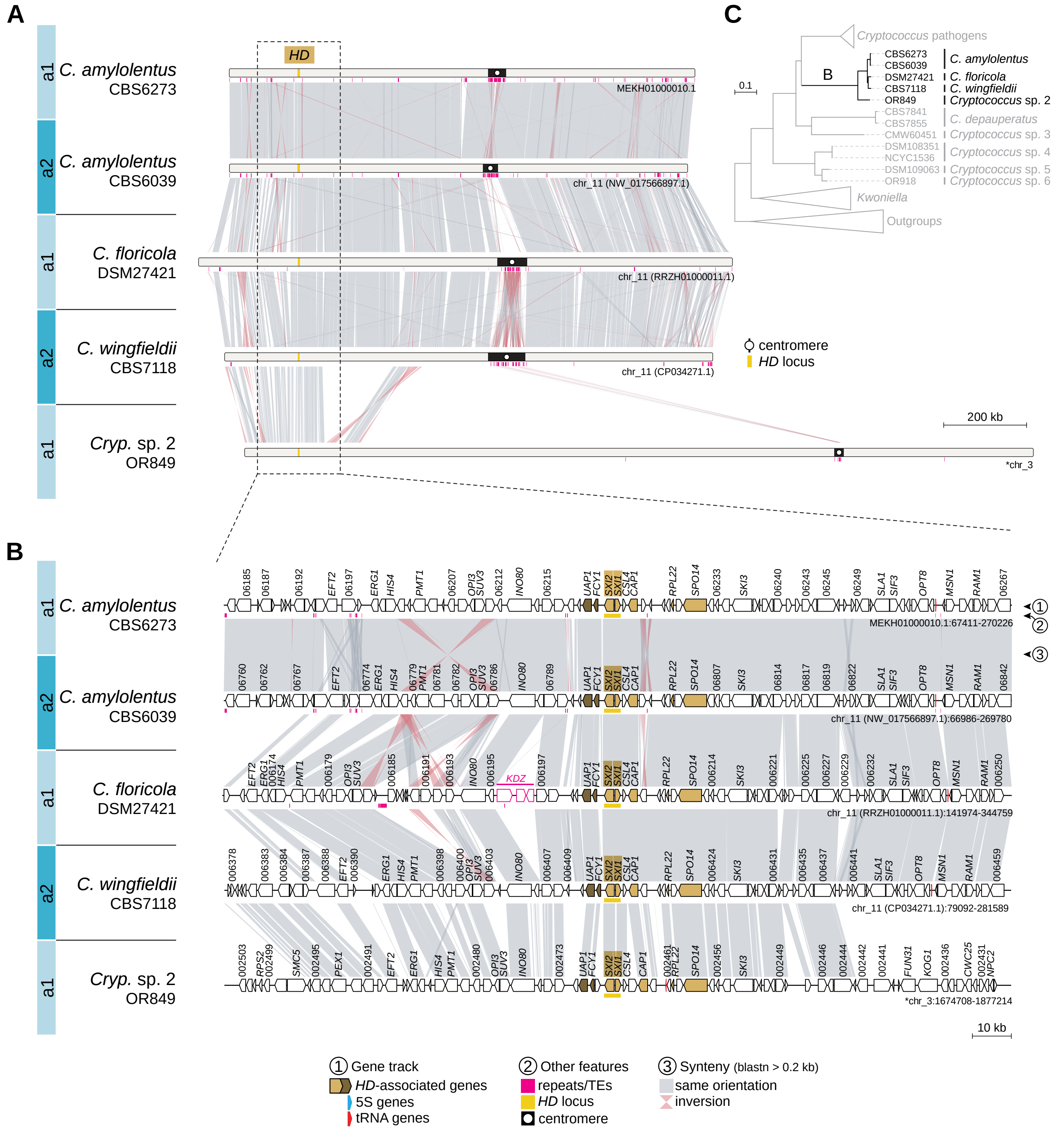

A
B
C
a1 C. amylolentus CBS6273
a2 C. amylolentus CBS6039
a1 C. floricola DSM27421
a2 C. wingfieldii CBS7118
a1 Cryp. sp. 2 OR849
HD
MEKH01000010.1
chr_11 (NW_017566897.1)
chr_11 (RRZH01000011.1)
chr_11 (CP034271.1)
*chr_3
centromere
HD locus
200 kb
Cryptococcus pathogens
CBS6273
CBS6039
DSM27421
CBS7118
OR849
CBS7841
CBS7855
CMW60451
DSM108351
NCYC1536
DSM109063
OR918
C. amylolentus
C. floricola
C. wingfieldii
Cryptococcus sp. 2
C. depauperatus
Cryptococcus sp. 3
Cryptococcus sp. 4
Cryptococcus sp. 5
Cryptococcus sp. 6
Kwoniella
Outgroups
0.1
MEKH01000010.1:67411-270226
chr_11 (NW_017566897.1):66986-269780
chr_11 (RRZH01000011.1):141974-344759
chr_11 (CP034271.1):79092-281589
*chr_3:1674708-1877214
KDZ
UAP1 FCY1 SXI2 SXI1 CSL4 CAP1
RPL22 SPO14 SKI3 SLA1 SIF3 OPT8 MSN1 RAM1
EFT2 ERG1 HIS4 PMT1 OPI3 SUV3 INO80
10 kb
① Gene track
HD-associated genes
5S genes
tRNA genes
② Other features
repeats/TEs
HD locus
centromere
③ Synteny (blastn > 0.2 kb)
same orientation
inversion

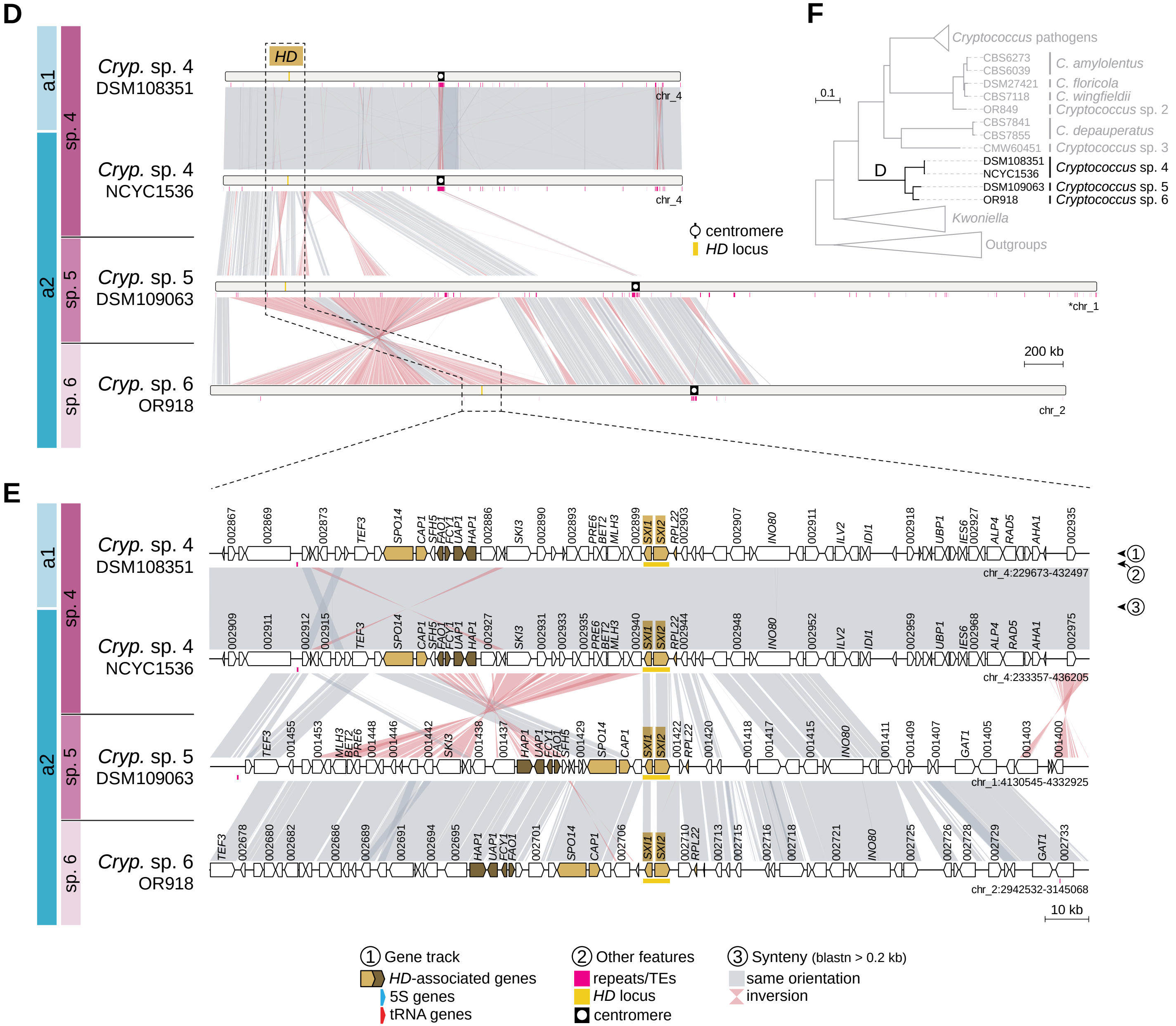

D
a1
a2
sp. 4
sp. 5
sp. 6
Cryp. sp. 4
DSM108351
Cryp. sp. 4
NCYC1536
Cryp. sp. 5
DSM109063
Cryp. sp. 6
OR918
HD
chr_4
chr_4
*chr_1
chr_2
centromere
HD locus
200 kb
F
Cryptococcus pathogens
CBS6273
CBS6039
DSM27421
CBS7118
OR849
CBS7841
CBS7855
CMW60451
DSM108351
NCYC1536
DSM109063
OR918
C. amylolentus
C. floricola
C. wingfieldii
Cryptococcus sp. 2
C. depauperatus
Cryptococcus sp. 3
Cryptococcus sp. 4
Cryptococcus sp. 5
Cryptococcus sp. 6
Kwoniella
Outgroups
0.1
E
chr_4:229673-432497
chr_4:233357-436205
chr_1:4130545-4332925
chr_2:2942532-3145068
10 kb
TEF3
SPO14
CAP1
SFH5
FAO1
FCY1
UAP1
HAP1
SKI3
PRE6
BET2
MLH3
SXI1
SXI2
RPL22
INO80
ILV2
IDI1
UBP1
IES6
ALP4
RAD5
AHA1
GAT1
① Gene track
HD-associated genes
5S genes
tRNA genes
② Other features
repeats/TEs
HD locus
centromere
③ Synteny (blastn > 0.2 kb)
same orientation
inversion

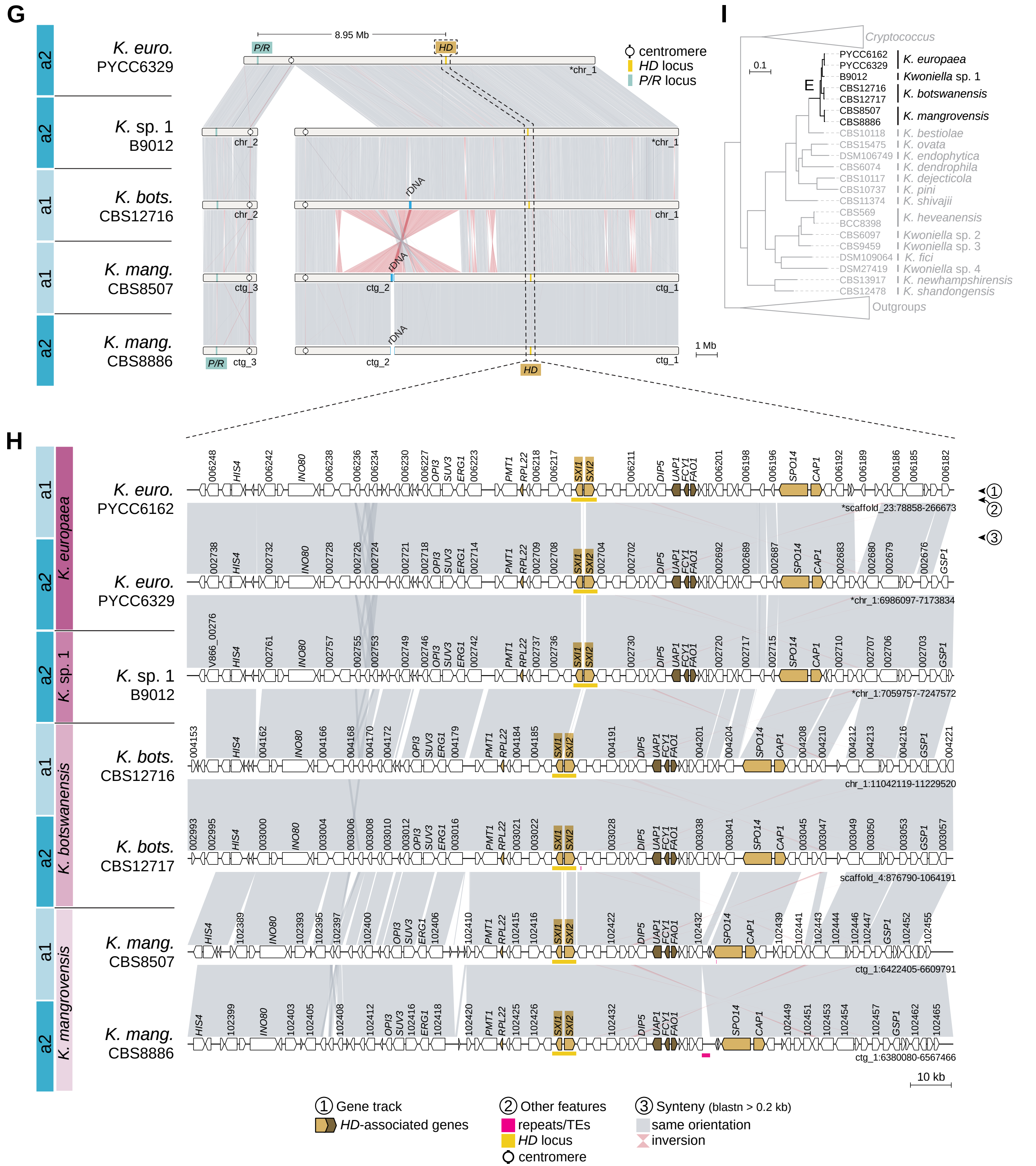

G
a2
K. euro.
PYCC6329
a2
K. sp. 1
B9012
a1
K. bots.
CBS12716
a1
K. mang.
CBS8507
a2
K. mang.
CBS8886
8.95 Mb
P/R
HD
*chr_1
chr_2
*chr_1
chr_2
chr_1
rDNA
ctg_3
ctg_2
ctg_1
rDNA
ctg_3
ctg_2
ctg_1
rDNA
P/R
HD
1 Mb
centromere
HD locus
P/R locus
I
Cryptococcus
0.1
E
PYCC6162
PYCC6329
K. europaea
B9012
Kwoniella sp. 1
CBS12716
CBS12717
K. botswanensis
CBS8507
CBS8886
K. mangrovensis
CBS10118
K. bestiolae
CBS15475
K. ovata
DSM106749
K. endophytica
CBS6074
K. dendrophila
CBS10117
K. dejecticola
CBS10737
K. pini
CBS11374
K. shivajii
CBS569
BCC8398
K. heveanensis
CBS6097
Kwoniella sp. 2
CBS9459
Kwoniella sp. 3
DSM109064
K. fici
DSM27419
Kwoniella sp. 4
CBS13917
K. newhampshirensis
CBS12478
K. shandongensis
Outgroups
H
a1
K. europaea
K. euro.
PYCC6162
*scaffold_23:78858-266673
a2
K. euro.
PYCC6329
*chr_1:6986097-7173834
a2
K. sp. 1
K. sp. 1
B9012
*chr_1:7059757-7247572
a1
K. botswanensis
K. bots.
CBS12716
chr_1:11042119-11229520
a2
K. bots.
CBS12717
scaffold_4:876790-1064191
a1
K. mangrovensis
K. mang.
CBS8507
ctg_1:6422405-6609791
a2
K. mang.
CBS8886
ctg_1:6380080-6567466
HIS4
INO80
OPI3
SUV3
ERG1
PMT1
RPL22
SXI1
SXI2
DIP5
UAP1
FCY1
FAO1
SPO14
CAP1
GSP1
10 kb
1
2
3
1 Gene track
HD-associated genes
2 Other features
repeats/TEs
HD locus
centromere
3 Synteny (blastn > 0.2 kb)
same orientation
inversion

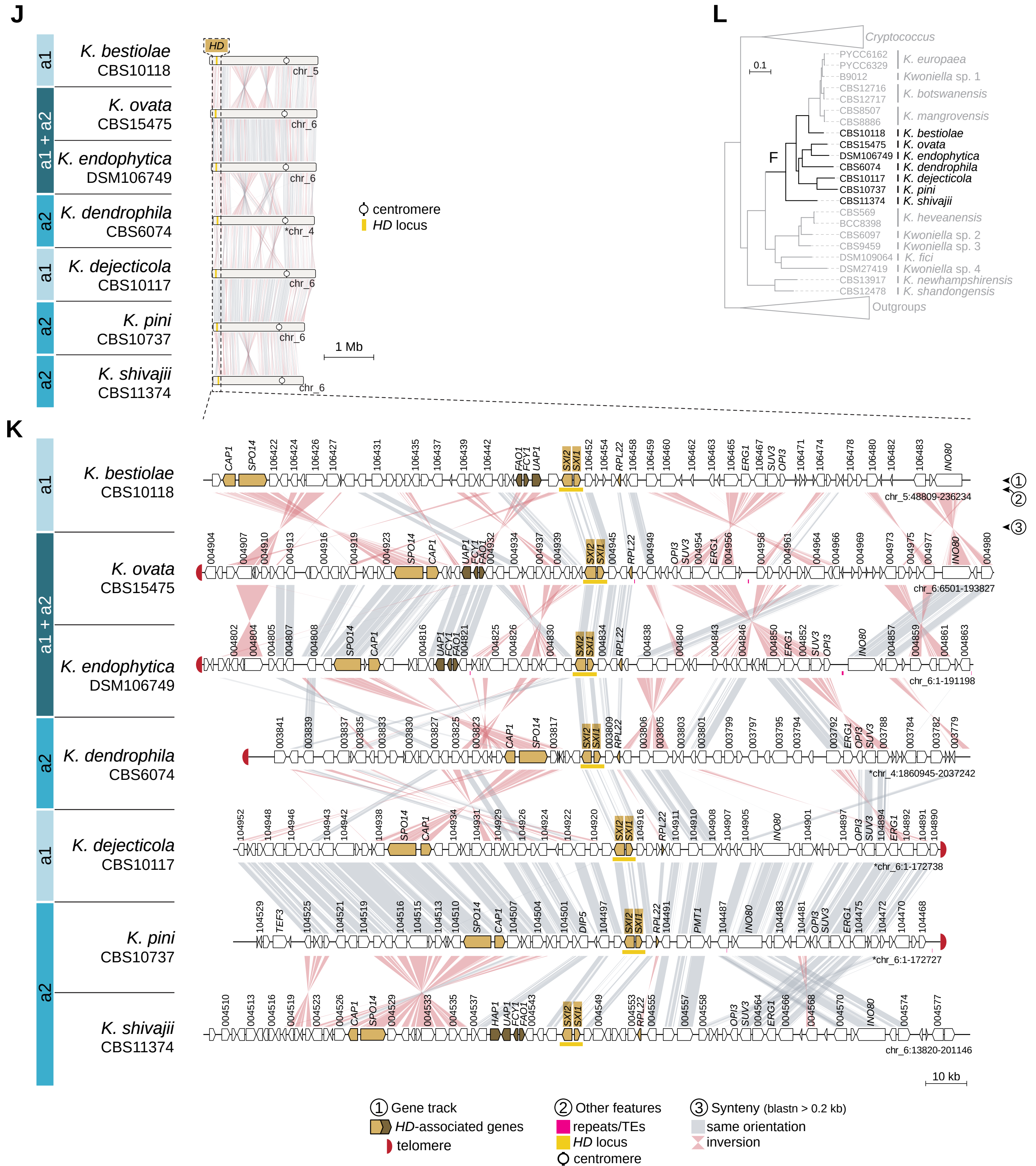
J
K
L
a1
a1 + a2
a2
K. bestiolae CBS10118
K. ovata CBS15475
K. endophytica DSM106749
K. dendrophila CBS6074
K. dejecticola CBS10117
K. pini CBS10737
K. shivajii CBS11374
HD
chr_5
chr_6
*chr_4
centromere
HD locus
1 Mb
chr_5:48809-236234
chr_6:6501-193827
chr_6:1-191198
*chr_4:1860945-2037242
*chr_6:1-172738
*chr_6:1-172727
chr_6:13820-201146
10 kb
CAP1
SPO14
FAO1
FCY1
UAP1
HAP1
SXI2
SXI1
RPL22
ERG1
SUV3
OPI3
INO80
TEF3
DIP5
PMT1
1 Gene track
HD-associated genes
telomere
2 Other features
repeats/TEs
HD locus
centromere
3 Synteny (blastn > 0.2 kb)
same orientation
inversion
Cryptococcus
0.1
PYCC6162
PYCC6329
K. europaea
B9012
Kwoniella sp. 1
CBS12716
CBS12717
K. botswanensis
CBS8507
CBS8886
K. mangrovensis
CBS10118
K. bestiolae
CBS15475
K. ovata
DSM106749
K. endophytica
CBS6074
K. dendrophila
CBS10117
K. dejecticola
CBS10737
K. pini
CBS11374
K. shivajii
F
CBS569
BCC8398
K. heveanensis
CBS6097
Kwoniella sp. 2
CBS9459
Kwoniella sp. 3
DSM109064
K. fici
DSM27419
Kwoniella sp. 4
CBS13917
K. newhampshirensis
CBS12478
K. shandongensis
Outgroups

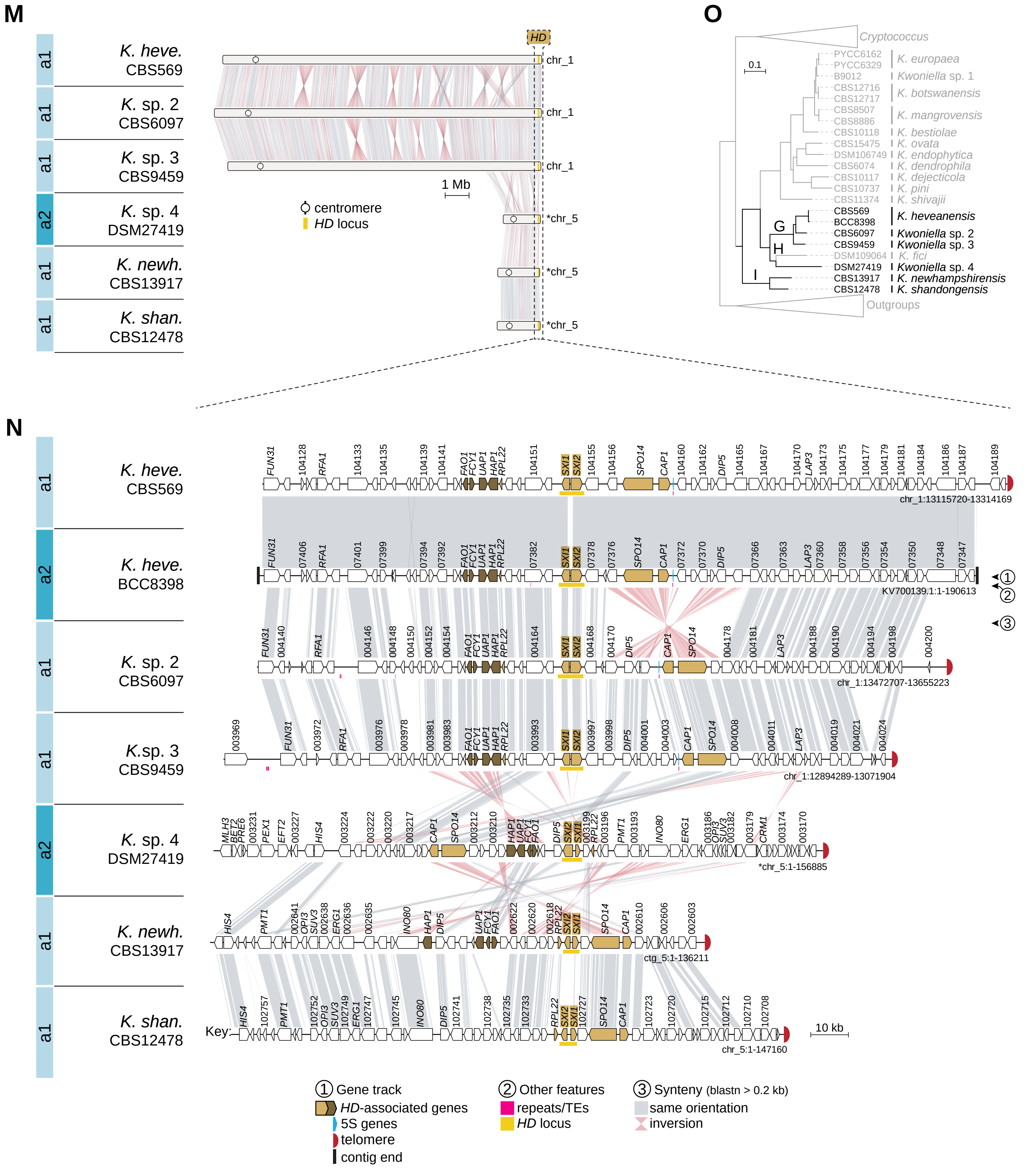
M
a1 K. heve. CBS569
a1 K. sp. 2 CBS6097
a1 K. sp. 3 CBS9459
a2 K. sp. 4 DSM27419
a1 K. newh. CBS13917
a1 K. shan. CBS12478
HD
chr_1
chr_1
chr_1
*chr_5
*chr_5
*chr_5
1 Mb
centromere
HD locus
O
Cryptococcus
0.1
PYCC6162
PYCC6329
K. europaea
B9012
Kwoniella sp. 1
CBS12716
CBS12717
K. botswanensis
CBS8507
CBS8886
K. mangrovensis
CBS10118
K. bestiolae
CBS15475
K. ovata
DSM106749
K. endophytica
CBS6074
K. dendrophila
CBS10117
K. dejecticola
CBS10737
K. pini
CBS11374
K. shivajii
CBS569
BCC8398
K. heveanensis
G
CBS6097
Kwoniella sp. 2
H
CBS9459
Kwoniella sp. 3
DSM109064
K. fici
I
DSM27419
Kwoniella sp. 4
CBS13917
K. newhampshirensis
CBS12478
K. shandongensis
Outgroups
N
a1 K. heve. CBS569
a2 K. heve. BCC8398
a1 K. sp. 2 CBS6097
a1 K.sp. 3 CBS9459
a2 K. sp. 4 DSM27419
a1 K. newh. CBS13917
a1 K. shan. CBS12478
SXI1
SXI2
SPO14
CAP1
FAO1
FCY1
UAP1
HAP1
RPL22
FUN31
RFA1
DIP5
LAP3
HIS4
PMT1
INO80
ERG1
chr_1:13115720-13314169
KV700139.1:1-190613
chr_1:13472707-13655223
chr_1:12894289-13071904
*chr_5:1-156885
ctg_5:1-136211
chr_5:1-147160
Key:
10 kb
1 Gene track
HD-associated genes
5S genes
telomere
contig end
2 Other features
repeats/TEs
HD locus
3 Synteny (blastn > 0.2 kb)
same orientation
inversion
