# Supplementary material for: Tracing the evolution and genomic dynamics of mating-type loci in Cryptococcus pathogens and closely related species
Source: bioRxiv. 2025 Aug 30:2025.02.12.637874. Originally published 2025 Feb 16. Preprint. [Version 2] doi: 10.1101/2025.02.12.637874 (PMC11844451; doi:10.1101/2025.02.12.637874)
Supplement: Supplement 4 — S4 Fig. Structure and genomic context of the P/R mating-type locus in tetrapolar Cryptococcus and Kwoniella species. This supplementary figure spans five pages and contains a total of 15 panels (labeled A–O), with three consecutive panels per page. For each group of three panels, the top-left panel shows a synteny view of the full chromosomes, highlighting the chromosomal location of the P/R locus; the bottom panel provides a zoomed-in synteny view (~200 kb) that includes the full P/R locus; and the top-right panel displays a simplified phylogenetic tree, providing contextual information for the species included in the synteny analyses. For cross-referencing, P/R-associated genes in the zoomed-in panel are colored teal if the corresponding ortholog in Cryptococcus pathogens is located within the MAT locus, shown in a darker shade when positioned in the flanking regions, or colored bright green if the gene is found within the P/R locus of some species. The P/R allele of each strain (a1 or a2) is indicated on the left. Chromosomes inverted relative to their original assembly orientations are marked with asterisks. In panels A and D, the GC content is depicted as the deviation from the genome average, calculated in 0.5 kb non-overlapping windows. In panel G, only one representative of K. europaea and K. botswanensis is shown, and in panel M, only one representative of K. heveanensis is included, as the genomes of their mating-type counterparts are not assembled at the chromosome level; the scaffolds containing the P/R locus are, however, included in the zoomed-in view. Other features are annotated as shown in the key. [file media-4.pdf]

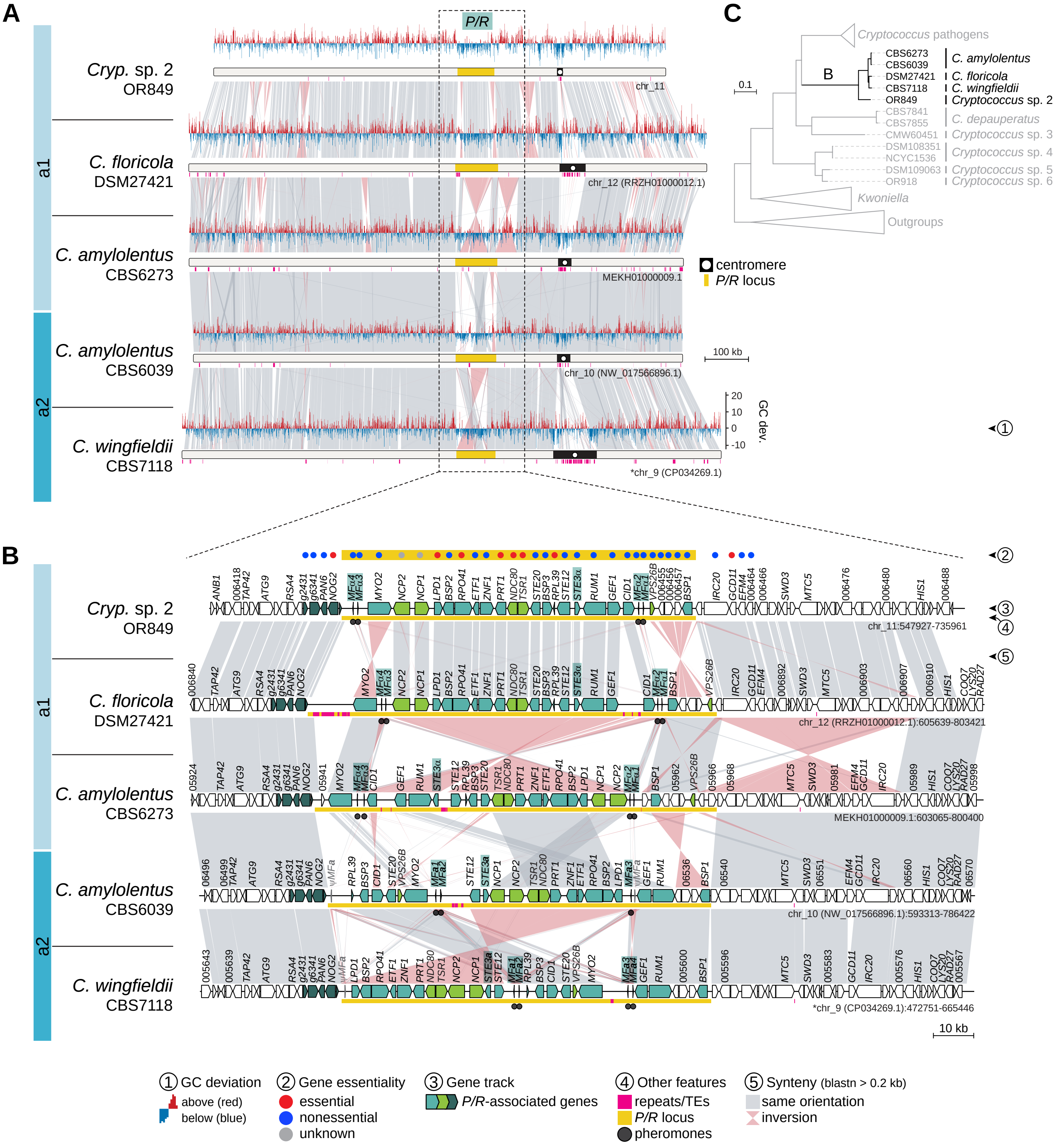

A
P/R
a1
a2
Cryp. sp. 2
OR849
chr_11
C. floricola
DSM27421
chr_12 (RRZH01000012.1)
C. amylolentus
CBS6273
MEKH01000009.1
C. amylolentus
CBS6039
chr_10 (NW_017566896.1)
C. wingfieldii
CBS7118
*chr_9 (CP034269.1)
centromere
P/R locus
100 kb
20
10
0
-10
GC dev.
C
Cryptococcus pathogens
CBS6273
CBS6039
DSM27421
CBS7118
OR849
C. amylolentus
C. floricola
C. wingfieldii
Cryptococcus sp. 2
0.1
B
CBS7841
CBS7855
C. depauperatus
CMW60451
Cryptococcus sp. 3
DSM108351
NCYC1536
Cryptococcus sp. 4
DSM109063
Cryptococcus sp. 5
OR918
Cryptococcus sp. 6
Kwoniella
Outgroups
B
chr_11:547927-735961
chr_12 (RRZH01000012.1):605639-803421
MEKH01000009.1:603065-800400
chr_10 (NW_017566896.1):593313-786422
*chr_9 (CP034269.1):472751-665446
10 kb
① GC deviation
above (red)
below (blue)
② Gene essentiality
essential
nonessential
unknown
③ Gene track
P/R-associated genes
④ Other features
repeats/TEs
P/R locus
pheromones
⑤ Synteny (blastn > 0.2 kb)
same orientation
inversion

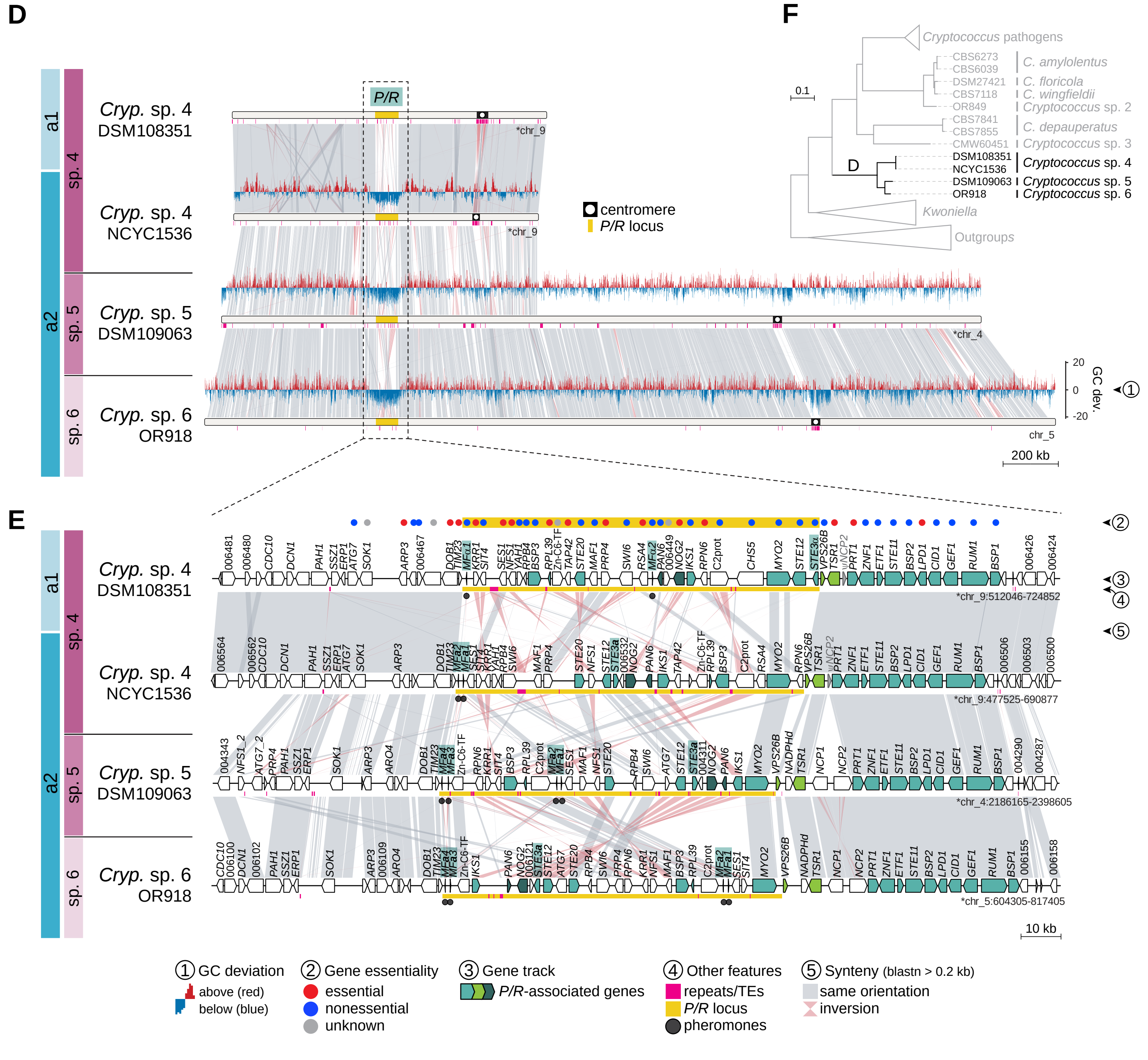

D
a1
a2
sp. 4
sp. 5
sp. 6
Cryp. sp. 4 DSM108351
Cryp. sp. 4 NCYC1536
Cryp. sp. 5 DSM109063
Cryp. sp. 6 OR918
P/R
*chr_9
*chr_4
chr_5
centromere
P/R locus
GC dev.
20
0
-20
200 kb
E
*chr_9:512046-724852
*chr_9:477525-690877
*chr_4:2186165-2398605
*chr_5:604305-817405
10 kb
F
Cryptococcus pathogens
CBS6273
CBS6039
DSM27421
CBS7118
OR849
CBS7841
CBS7855
CMW60451
DSM108351
NCYC1536
DSM109063
OR918
C. amylolentus
C. floricola
C. wingfieldii
Cryptococcus sp. 2
C. depauperatus
Cryptococcus sp. 3
Cryptococcus sp. 4
Cryptococcus sp. 5
Cryptococcus sp. 6
Kwoniella
Outgroups
0.1
① GC deviation
above (red)
below (blue)
② Gene essentiality
essential
nonessential
unknown
③ Gene track
P/R-associated genes
④ Other features
repeats/TEs
P/R locus
pheromones
⑤ Synteny (blastn > 0.2 kb)
same orientation
inversion

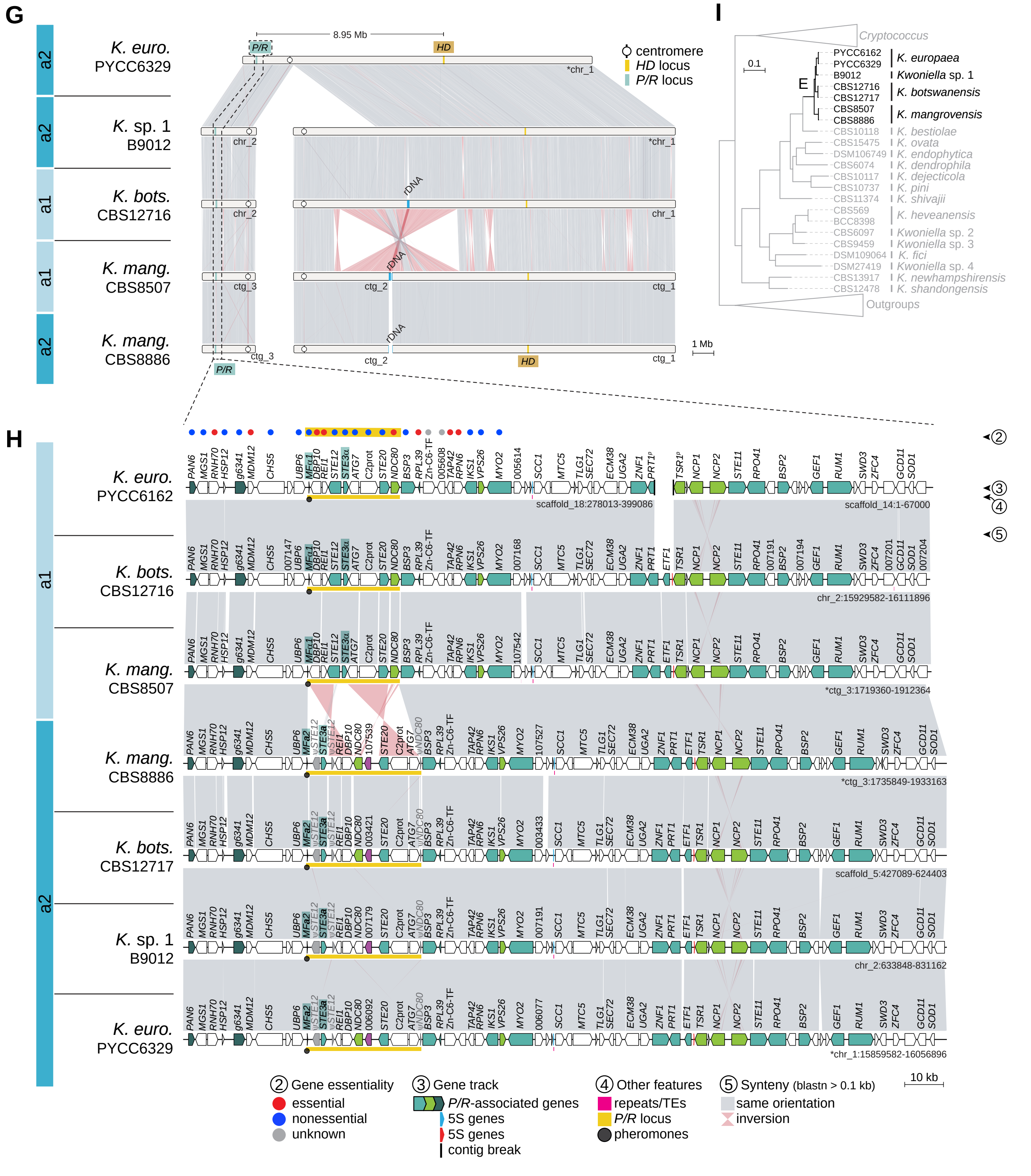
G
a2
K. euro.
PYCC6329
a2
K. sp. 1
B9012
a1
K. bots.
CBS12716
a1
K. mang.
CBS8507
a2
K. mang.
CBS8886
8.95 Mb
P/R
HD
*chr_1
chr_2
*chr_1
chr_2
chr_1
rDNA
ctg_3
ctg_2
ctg_1
rDNA
ctg_3
ctg_2
ctg_1
rDNA
P/R
HD
1 Mb
centromere
HD locus
P/R locus
I
Cryptococcus
0.1
E
PYCC6162
PYCC6329
B9012
CBS12716
CBS12717
CBS8507
CBS8886
CBS10118
CBS15475
DSM106749
CBS6074
CBS10117
CBS10737
CBS11374
CBS569
BCC8398
CBS6097
CBS9459
DSM109064
DSM27419
CBS13917
CBS12478
K. europaea
Kwoniella sp. 1
K. botswanensis
K. mangrovensis
K. bestiolae
K. ovata
K. endophytica
K. dendrophila
K. dejecticola
K. pini
K. shivajii
K. heveanensis
Kwoniella sp. 2
Kwoniella sp. 3
K. fici
Kwoniella sp. 4
K. newhampshirensis
K. shandongensis
Outgroups
H
a1
K. euro.
PYCC6162
K. bots.
CBS12716
K. mang.
CBS8507
a2
K. mang.
CBS8886
K. bots.
CBS12717
K. sp. 1
B9012
K. euro.
PYCC6329
PAN6 MGS1 RNH70 HSP12 g6341 MDM12 CHS5 UBP6 MFα1 DBP10 REI1 STE12 STE3α ATG7 C2prot STE20 NDC80 BSP3 RPL39 Zn-C6-TF 005608 TAP42 RPN6 IKS1 VPS26 MYO2 005614 SCC1 MTC5 TLG1 SEC72 ECM38 UGA2 ZNF1 PRT1p TSR1p NCP1 NCP2 STE11 RPO41 BSP2 GEF1 RUM1 SWD3 ZFC4 GCD11 SOD1
scaffold_18:278013-399086
scaffold_14:1-67000
PAN6 MGS1 RNH70 HSP12 g6341 MDM12 CHS5 007147 UBP6 MFα1 DBP10 REI1 STE12 STE3α ATG7 C2prot STE20 NDC80 BSP3 RPL39 Zn-C6-TF TAP42 RPN6 IKS1 VPS26 MYO2 007168 SCC1 MTC5 TLG1 SEC72 ECM38 UGA2 ZNF1 PRT1 ETF1 TSR1 NCP1 NCP2 STE11 RPO41 007191 BSP2 007194 GEF1 RUM1 SWD3 ZFC4 007201 GCD11 SOD1 007204
chr_2:15929582-16111896
PAN6 MGS1 RNH70 HSP12 g6341 MDM12 CHS5 UBP6 MFα1 DBP10 REI1 STE12 STE3α ATG7 C2prot STE20 NDC80 BSP3 RPL39 Zn-C6-TF TAP42 RPN6 IKS1 VPS26 MYO2 107542 SCC1 MTC5 TLG1 SEC72 ECM38 UGA2 ZNF1 PRT1 ETF1 TSR1 NCP1 NCP2 STE11 RPO41 BSP2 GEF1 RUM1 SWD3 ZFC4 GCD11 SOD1
*ctg_3:1719360-1912364
PAN6 MGS1 RNH70 HSP12 g6341 MDM12 CHS5 UBP6 MFa2 ψSTE12 STE3a ψSTE12 REI1 DBP10 NDC80 107539 STE20 C2prot ATG7 ψNDC80 BSP3 RPL39 Zn-C6-TF TAP42 RPN6 IKS1 VPS26 MYO2 107527 SCC1 MTC5 TLG1 SEC72 ECM38 UGA2 ZNF1 PRT1 ETF1 TSR1 NCP1 NCP2 STE11 RPO41 BSP2 GEF1 RUM1 SWD3 ZFC4 GCD11 SOD1
*ctg_3:1735849-1933163
PAN6 MGS1 RNH70 HSP12 g6341 MDM12 CHS5 UBP6 MFa2 ψSTE12 STE3a ψSTE12 REI1 DBP10 NDC80 003421 STE20 C2prot ATG7 ψNDC80 BSP3 RPL39 Zn-C6-TF TAP42 RPN6 IKS1 VPS26 MYO2 003433 SCC1 MTC5 TLG1 SEC72 ECM38 UGA2 ZNF1 PRT1 ETF1 TSR1 NCP1 NCP2 STE11 RPO41 BSP2 GEF1 RUM1 SWD3 ZFC4 GCD11 SOD1
scaffold_5:427089-624403
PAN6 MGS1 RNH70 HSP12 g6341 MDM12 CHS5 UBP6 MFa2 ψSTE12 STE3a ψSTE12 REI1 DBP10 NDC80 007179 STE20 C2prot ATG7 ψNDC80 BSP3 RPL39 Zn-C6-TF TAP42 RPN6 IKS1 VPS26 MYO2 007191 SCC1 MTC5 TLG1 SEC72 ECM38 UGA2 ZNF1 PRT1 ETF1 TSR1 NCP1 NCP2 STE11 RPO41 BSP2 GEF1 RUM1 SWD3 ZFC4 GCD11 SOD1
chr_2:633848-831162
PAN6 MGS1 RNH70 HSP12 g6341 MDM12 CHS5 UBP6 MFa2 ψSTE12 STE3a ψSTE12 REI1 DBP10 NDC80 006092 STE20 C2prot ATG7 ψNDC80 BSP3 RPL39 Zn-C6-TF TAP42 RPN6 IKS1 VPS26 MYO2 006077 SCC1 MTC5 TLG1 SEC72 ECM38 UGA2 ZNF1 PRT1 ETF1 TSR1 NCP1 NCP2 STE11 RPO41 BSP2 GEF1 RUM1 SWD3 ZFC4 GCD11 SOD1
*chr_1:15859582-16056896
10 kb
② Gene essentiality
essential
nonessential
unknown
③ Gene track
P/R-associated genes
5S genes
5S genes
contig break
④ Other features
repeats/TEs
P/R locus
pheromones
⑤ Synteny (blastn > 0.1 kb)
same orientation
inversion

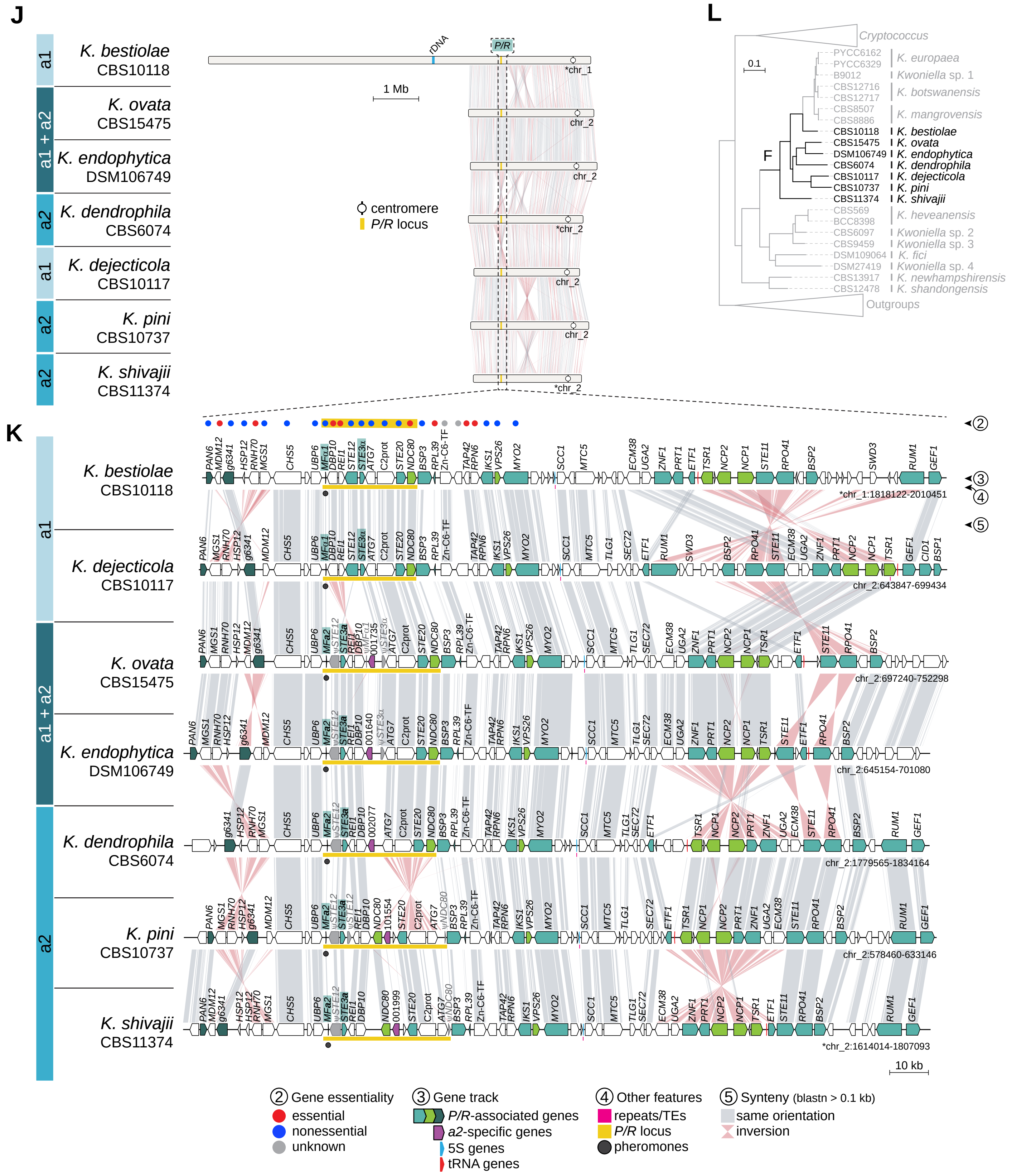

J
K. bestiolae CBS10118
K. ovata CBS15475
K. endophytica DSM106749
K. dendrophila CBS6074
K. dejecticola CBS10117
K. pini CBS10737
K. shivajii CBS11374
a1
a1 + a2
a2
rDNA
P/R
1 Mb
*chr_1
chr_2
*chr_2
centromere
P/R locus
L
Cryptococcus
0.1
PYCC6162
PYCC6329
K. europaea
B9012
Kwoniella sp. 1
CBS12716
CBS12717
K. botswanensis
CBS8507
CBS8886
K. mangrovensis
CBS10118 K. bestiolae
CBS15475 K. ovata
DSM106749 K. endophytica
CBS6074 K. dendrophila
CBS10117 K. dejecticola
CBS10737 K. pini
CBS11374 K. shivajii
F
CBS569
BCC8398
K. heveanensis
CBS6097 Kwoniella sp. 2
CBS9459 Kwoniella sp. 3
DSM109064 K. fici
DSM27419 Kwoniella sp. 4
CBS13917 K. newhampshirensis
CBS12478 K. shandongensis
Outgroups
K
*chr_1:1818122-2010451
chr_2:643847-699434
chr_2:697240-752298
chr_2:645154-701080
chr_2:1779565-1834164
chr_2:578460-633146
*chr_2:1614014-1807093
10 kb
PAN6 MDM12 g6341 HSP12 RNH70 MGS1 CHS5 UBP6 MFα1 DBP10 REI1 STE12 STE3α ATG7 C2prot STE20 NDC80 BSP3 RPL39 Zn-C6-TF TAP42 RPN6 IKS1 VPS26 MYO2 SCC1 MTC5 ECM38 UGA2 ZNF1 PRT1 ETF1 TSR1 NCP2 NCP1 STE11 RPO41 BSP2 SWD3 RUM1 GEF1
TLG1 SEC72 CID1
MFa2 STE3a ψSTE12 ψMFα1 ψSTE3α 001735 001640 002077 101554 ψNDC80 001999
② Gene essentiality
essential
nonessential
unknown
③ Gene track
P/R-associated genes
a2-specific genes
5S genes
tRNA genes
④ Other features
repeats/TEs
P/R locus
pheromones
⑤ Synteny (blastn > 0.1 kb)
same orientation
inversion

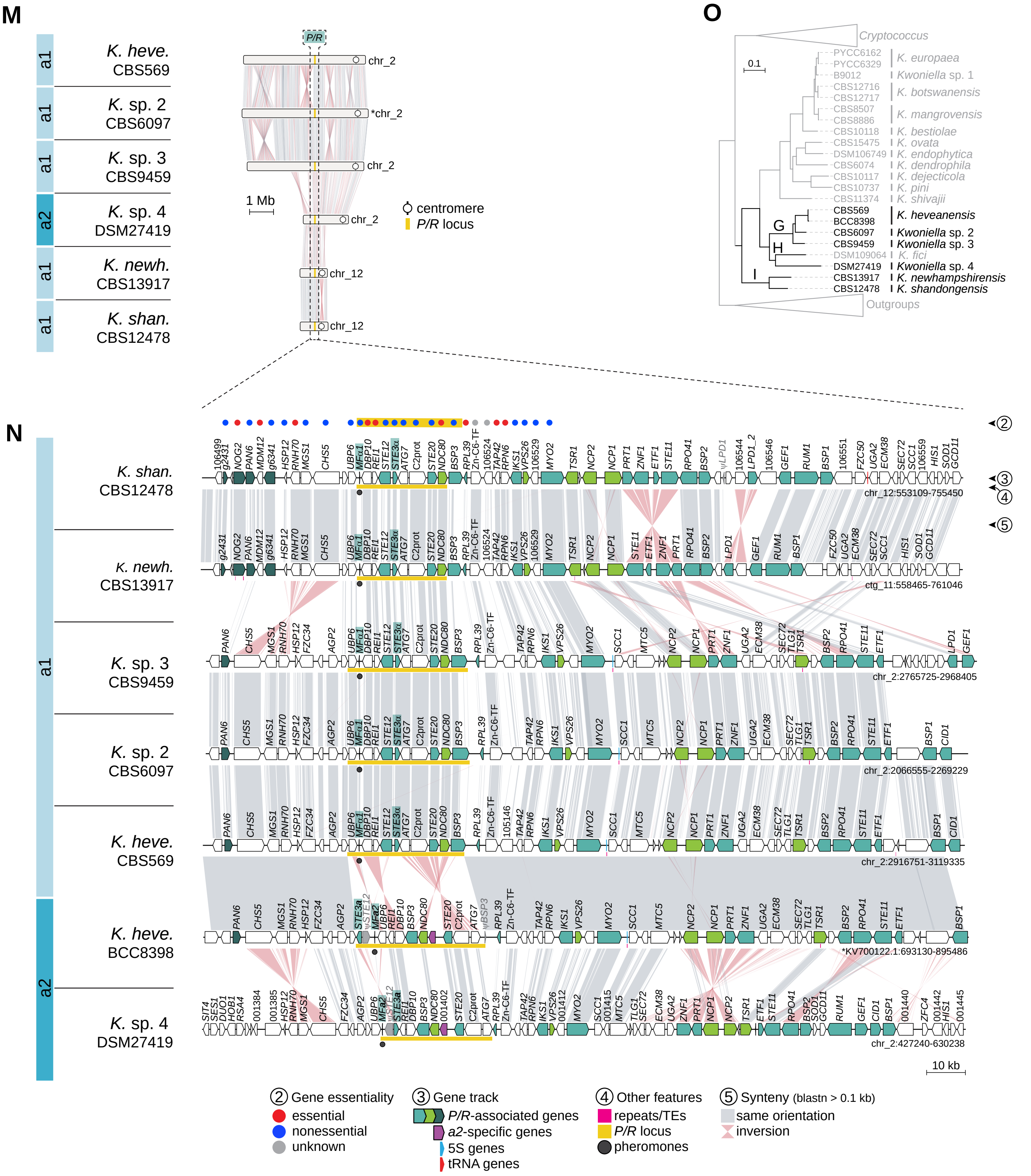

M
O
N
K. heve. CBS569
K. sp. 2 CBS6097
K. sp. 3 CBS9459
K. sp. 4 DSM27419
K. newh. CBS13917
K. shan. CBS12478
P/R
1 Mb
centromere
P/R locus
Cryptococcus
Outgroups
K. europaea
Kwoniella sp. 1
K. botswanensis
K. mangrovensis
K. bestiolae
K. ovata
K. endophytica
K. dendrophila
K. dejecticola
K. pini
K. shivajii
K. heveanensis
Kwoniella sp. 2
Kwoniella sp. 3
K. fici
Kwoniella sp. 4
K. newhampshirensis
K. shandongensis
K. heve. BCC8398
chr_12:553109-755450
ctg_11:558465-761046
chr_2:2765725-2968405
chr_2:2066555-2269229
chr_2:2916751-3119335
*KV700122.1:693130-895486
chr_2:427240-630238
10 kb
2 Gene essentiality
essential
nonessential
unknown
3 Gene track
P/R-associated genes
a2-specific genes
5S genes
tRNA genes
4 Other features
repeats/TEs
P/R locus
pheromones
5 Synteny (blastn > 0.1 kb)
same orientation
inversion
