# Supplementary material for: Tracing the evolution and genomic dynamics of mating-type loci in Cryptococcus pathogens and closely related species
Source: bioRxiv. 2025 Aug 30:2025.02.12.637874. Originally published 2025 Feb 16. Preprint. [Version 2] doi: 10.1101/2025.02.12.637874 (PMC11844451; doi:10.1101/2025.02.12.637874)
Supplement: Supplement 6 — S6 Fig. MAT locus structure in Cryptococcus deneoformans. Synteny analysis is shown for five strains organized by mating type. Strain JEC20a is an F1 progeny of the cross between isolates NIH12α and NIH433a, while JEC21α is its congenic partner, generated through 10 rounds of backcrossing to JEC20a. Therefore, the MATα allele in JEC21α was inherited from NIH12α, and the MATa allele in JEC20a was inherited from NIH433a. The genes BSP3 and IKS1 are no longer part of the MAT locus in this species. Chromosomes inverted relative to their original assembly orientations are marked with asterisks. [file media-6.pdf]

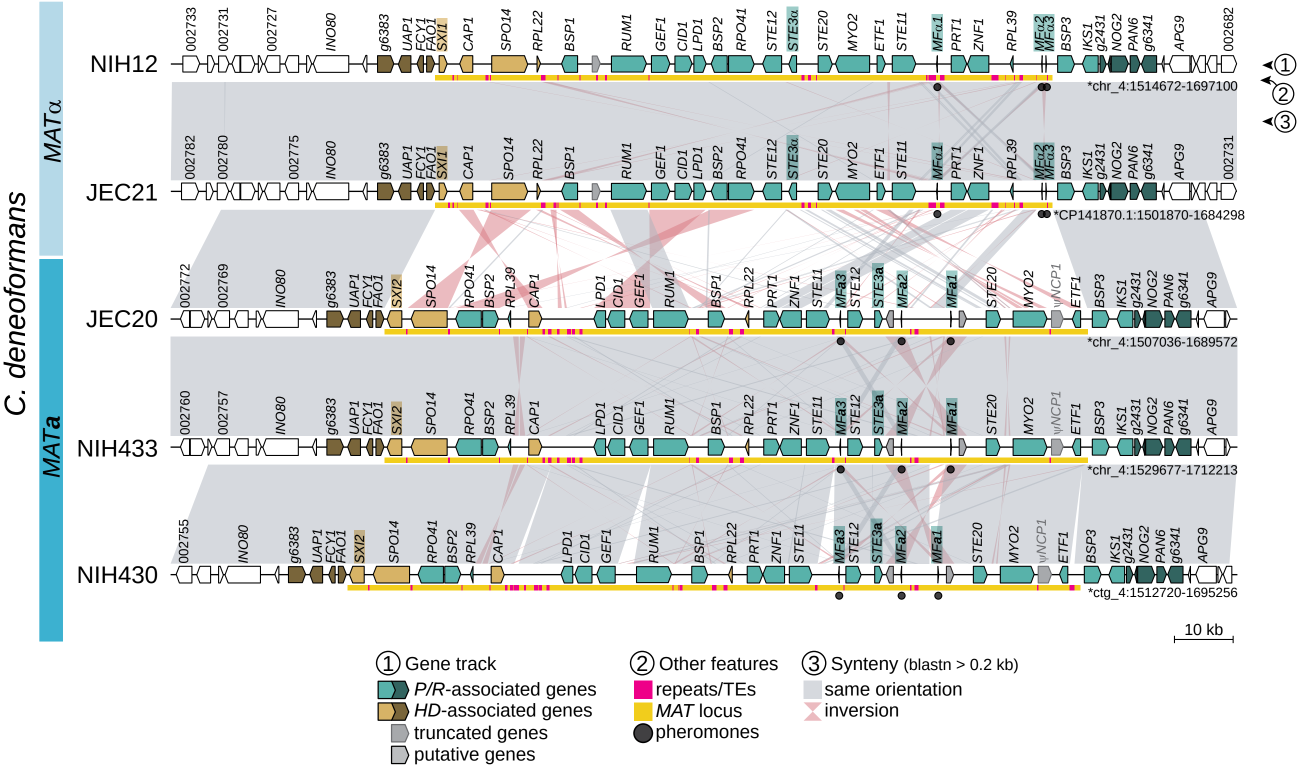

C. deneoformans
MATα
MATa
NIH12
JEC21
JEC20
NIH433
NIH430
*chr_4:1514672-1697100
*CP141870.1:1501870-1684298
*chr_4:1507036-1689572
*chr_4:1529677-1712213
*ctg_4:1512720-1695256
10 kb
① Gene track
P/R-associated genes
HD-associated genes
truncated genes
putative genes
② Other features
repeats/TEs
MAT locus
pheromones
③ Synteny (blastn > 0.2 kb)
same orientation
inversion
