# Supplementary material for: Tracing the evolution and genomic dynamics of mating-type loci in Cryptococcus pathogens and closely related species
Source: bioRxiv. 2025 Aug 30:2025.02.12.637874. Originally published 2025 Feb 16. Preprint. [Version 2] doi: 10.1101/2025.02.12.637874 (PMC11844451; doi:10.1101/2025.02.12.637874)
Supplement: Supplement 11 — S11 Fig. FACS analysis of K. mangrovensis parental strains CBS8507 and CBS10435, and their progeny. The C. neoformans strain JEC21 and XL143 served as haploid (1n), and diploid (2n) controls, respectively. [file media-11.pdf]

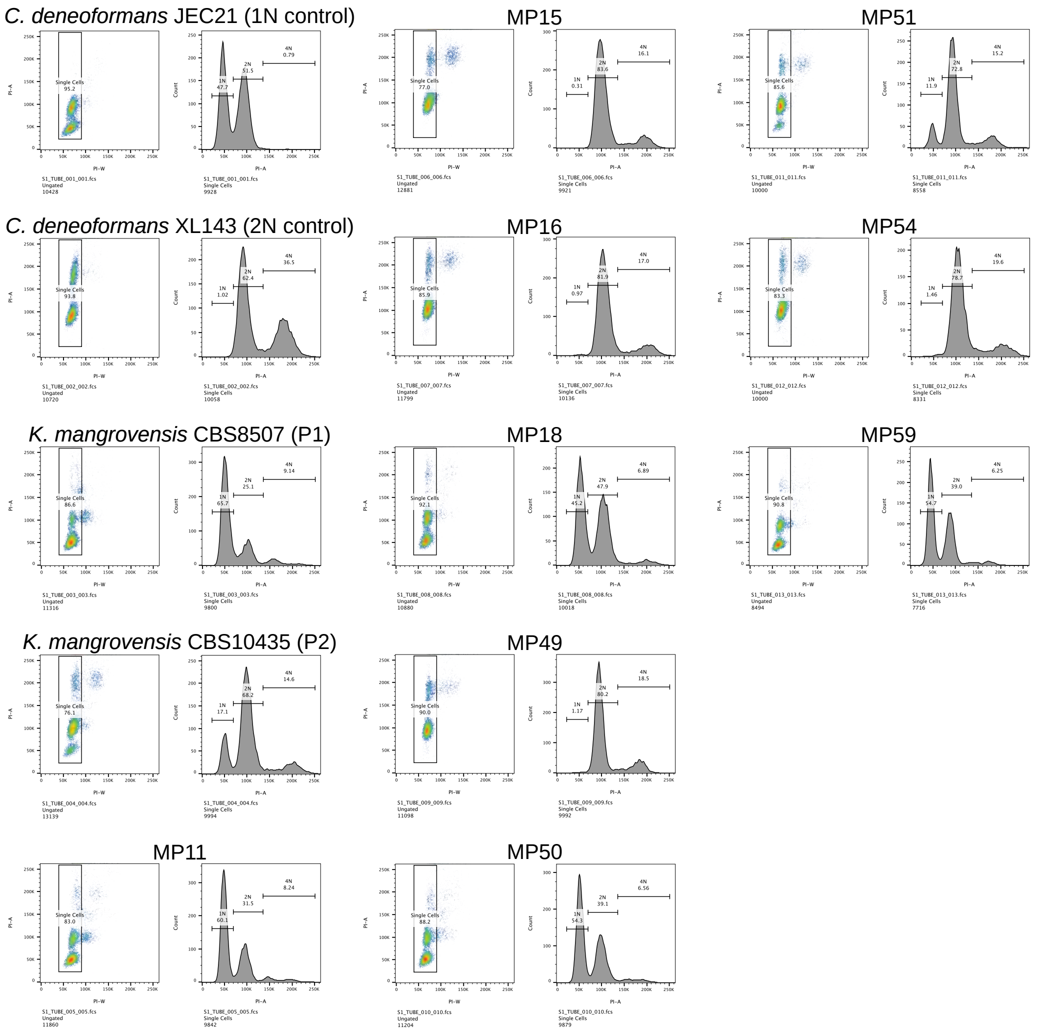
C. deneoformans JEC21 (1N control)
Single Cells 95.2
1N 47.7
2N 51.5
4N 0.79
S1_TUBE_001_001.fcs Ungated 10428
S1_TUBE_001_001.fcs Single Cells 9928
MP15
Single Cells 77.0
1N 0.31
2N 83.6
4N 16.1
S1_TUBE_006_006.fcs Ungated 12881
S1_TUBE_006_006.fcs Single Cells 9921
MP51
Single Cells 85.6
1N 11.9
2N 72.8
4N 15.2
S1_TUBE_011_011.fcs Ungated 10000
S1_TUBE_011_011.fcs Single Cells 8558
C. deneoformans XL143 (2N control)
Single Cells 93.8
1N 1.02
2N 62.4
4N 36.5
S1_TUBE_002_002.fcs Ungated 10720
S1_TUBE_002_002.fcs Single Cells 10058
MP16
Single Cells 85.9
1N 0.97
2N 81.9
4N 17.0
S1_TUBE_007_007.fcs Ungated 11799
S1_TUBE_007_007.fcs Single Cells 10136
MP54
Single Cells 83.3
1N 1.46
2N 78.7
4N 19.6
S1_TUBE_012_012.fcs Ungated 10000
S1_TUBE_012_012.fcs Single Cells 8331
K. mangrovensis CBS8507 (P1)
Single Cells 86.6
1N 65.7
2N 25.1
4N 9.14
S1_TUBE_003_003.fcs Ungated 11316
S1_TUBE_003_003.fcs Single Cells 9800
MP18
Single Cells 92.1
1N 45.2
2N 47.9
4N 6.89
S1_TUBE_008_008.fcs Ungated 10880
S1_TUBE_008_008.fcs Single Cells 10018
MP59
Single Cells 90.8
1N 54.7
2N 39.0
4N 6.25
S1_TUBE_013_013.fcs Ungated 8494
S1_TUBE_013_013.fcs Single Cells 7716
K. mangrovensis CBS10435 (P2)
Single Cells 76.1
1N 17.1
2N 68.2
4N 14.6
S1_TUBE_004_004.fcs Ungated 13139
S1_TUBE_004_004.fcs Single Cells 9994
MP49
Single Cells 90.0
1N 1.17
2N 80.2
4N 18.5
S1_TUBE_009_009.fcs Ungated 11098
S1_TUBE_009_009.fcs Single Cells 9992
MP11
Single Cells 83.0
1N 60.1
2N 31.5
4N 8.24
S1_TUBE_005_005.fcs Ungated 11860
S1_TUBE_005_005.fcs Single Cells 9842
MP50
Single Cells 88.2
1N 54.3
2N 39.1
4N 6.56
S1_TUBE_010_010.fcs Ungated 11204
S1_TUBE_010_010.fcs Single Cells 9879
