# Supplementary material for: Tracing the evolution and genomic dynamics of mating-type loci in Cryptococcus pathogens and closely related species
Source: bioRxiv. 2025 Aug 30:2025.02.12.637874. Originally published 2025 Feb 16. Preprint. [Version 2] doi: 10.1101/2025.02.12.637874 (PMC11844451; doi:10.1101/2025.02.12.637874)
Supplement: Supplement 14 — S2 Text. Karyotype reduction in Cryptococcus sp. 3 is independent of P/R–HD linkage. [file media-14.pdf]

S2 Text

We considered the possibility that the fusion of the *P/R* and *HD* loci in *Cryptococcus* sp. 3 might be a consequence of a reduction in chromosome number, since this species possesses 13 chromosomes compared to the inferred ancestral 14-chromosome karyotype [1]. Under this scenario, linkage of the two *MAT* loci could have resulted from a chromosomal fusion between the ancestral *P/R*- and *HD*-bearing chromosomes, coincident with the loss of one centromere.

To evaluate this hypothesis, we first identified which centromere was lost in *Cryptococcus* sp. 3, strain CWM60451. Centromere positions were inferred computationally based on the presence of characteristic long terminal repeat (LTR) elements typically enriched at *Cryptococcus* centromeres, in combination with synteny comparisons and identification of the longest ORF-free intergenic regions [1-4]. These predictions were then cross-validated by synteny comparisons with closely related species. Using this approach, we traced the missing centromere to chr. 3 of *Cryptococcus* sp. 3, which corresponds to the *CEN4* region of *C. neoformans* H99 (**S9B Fig**). In *Cryptococcus* sp. 3, this region no longer exhibits the characteristic LTR-rich structure typical of functional centromeres, consistent with centromere inactivation and eventual loss. Comparative synteny analyses with *K. shandongensis* further suggest that this loss could have arisen through intercentromeric recombination, in which the fusion of two centromeres led to the inactivation of one and stabilization of the chromosome with a single functional centromere.

Importantly, the *MAT* locus in *Cryptococcus* sp. 3 is located on chr. 11 (**Fig 6A** and **S9 Fig**), which is unrelated to the chromosome where centromere loss occurred. Thus, the reduction from 14 to 13 chromosomes in this lineage cannot account for the colocation of *P/R* and *HD*. Instead, our results indicate that physical linkage of the two *MAT* loci in *Cryptococcus* sp. 3 must have arisen through a separate structural event, independent of karyotype reduction.

References

1. Coelho MA, David-Palma M, Shea T, Bowers K, McGinley-Smith S, Mohammad AW, et al. Comparative genomics of the closely related fungal genera *Cryptococcus* and *Kwoniella* reveals karyotype dynamics and suggests evolutionary mechanisms of pathogenesis. PLOS Biol. 2024;22(6):e3002682. Epub 2024/06/06.
2. Janbon G, Ormerod KL, Paulet D, Byrnes EJ, 3rd, Yadav V, Chatterjee G, et al. Analysis of the genome and transcriptome of *Cryptococcus neoformans* var. *grubii* reveals complex RNA expression and microevolution leading to virulence attenuation. PLOS Genet. 2014;10(4):e1004261. Epub 2014/04/20.
3. Sun S, Yadav V, Billmyre RB, Cuomo CA, Nowrousian M, Wang L, et al. Fungal genome and mating system transitions facilitated by chromosomal translocations involving intercentromeric recombination. PLOS Biol. 2017;15(8):e2002527. Epub 2017/08/12.
4. Yadav V, Sun S, Billmyre RB, Thimmappa BC, Shea T, Lintner R, et al. RNAi is a critical determinant of centromere evolution in closely related fungi. Proc Natl Acad Sci U S A. 2018. Epub 2018/03/07.
